# Identification of olfactory genes and functional analysis of *BminCSP* and *BminOBP21* in *Bactrocera minax*

**DOI:** 10.1371/journal.pone.0222193

**Published:** 2019-09-11

**Authors:** Penghui Xu, Yaohui Wang, Mazarin Akami, Chang-Ying Niu

**Affiliations:** Department of Plant Protection, College of Plant Science & Technology, Huazhong Agricultural University, Wuhan, China; Chinese Academy of Agricultural Sciences Institute of Plant Protection, CHINA

## Abstract

Insects possess highly developed olfactory systems which play pivotal roles in its ecological adaptations, host plant location, and oviposition behavior. *Bactrocera minax* is an oligophagous tephritid insect whose host selection, and oviposition behavior largely depend on the perception of chemical cues. However, there have been very few reports on molecular components related to the olfactory system of *B*. *minax*. Therefore, the transcriptome of *B*. *minax* were sequenced in this study, with 1 candidate chemosensory protein (CSP), 21 candidate odorant binding proteins (OBPs), 53 candidate odorant receptors (ORs), 29 candidate ionotropic receptors (IRs) and 4 candidate sensory neuron membrane proteins (SNMPs) being identified. After that, we sequenced the candidate olfactory genes and performed phylogenetic analysis. qRT-PCR was used to express and characterize 9 genes in olfactory and non-olfactory tissues. Compared with GFP-injected fly (control), dsOBP21-treated *B*. *minax* and dsCSP-treated *B*. *minax* had lower electrophysiological response to D-limonene (attractant), suggesting the potential involvement of *BminOBP21* and *BminCSP* genes in olfactory perceptions of the fly. Our study establishes the molecular basis of olfaction, tributary for further functional analyses of chemosensory processes in *B*. *minax*.

## Introduction

Many research workers have pointed out that biosynthesis and receptor molecular recognition systems evolve in synchronous steps during animal signaling process [1–3]. Odors are a potential tool to control agricultural beneficial and injurious insects [4]. With highly sophisticated olfactory system insects can recognize various volatile chemicals from their prey, host plants and conspecifics [5, 6].

Sensory inputs can be converted into behavioral outputs by synaptic connections in highly streamlined olfactory circuits [7]. Antennae and maxillary palps are two important olfactory organs in the detection of olfactory signals and cues [8]. These organs are covered in sensilla that contain the dendrites of stereotypical combinations of olfactory sensory neurons (OSNs), odorant receptor (OR) or ionotropic receptor (IR) [9]. Normally, ORs are expressed in company with a co-receptor, which is called Orco [4, 10]. Compared with ORs, Orco is widely expressed in olfactory sensory neurons and plays a vital role in olfactory transduction [7, 11]. IR families can be categorized into three subgroups, including “antennal IRs” “divergent IRs” and iGluRs. iGluRs and “antennal IRs” which are extensively expressed in coeloconic OSNs of antenna [12]. Odorant binding proteins (OBPs) and chemosensory proteins (CSPs) are typically located on antennae and mouthparts and are also major proteins involved in recognition of volatiles. OBPs and CSPs play an important role in transporting incoming odorants to corresponding receptors and in transferring the odorant-degrading enzymes (ODEs) to the receptors [13, 14]. Previous studies have shown that insects communicate with their environment through detection of odorant molecules [15]. The olfactory systems of insects are highly selective for semiochemicals, which are of great importance to the mediation of their behavior patterns such as location of mates and food sources [16, 17]. Therefore, investigating the gene function in semiochemical detection is an essential step towards understanding the mechanism of olfaction in insects.

As a univoltine, oligophagous tephritid, *Bactrocera minax* is mainly distributed in the citrus production areas of China, India and Bhutan [18, 19]. The adult female oviposits and larvae develop primarily in citrus. After hatching, larvae is fed and protected within the reproductive structures of the host plant until completion of their larval stage [20]. The endophytic behavior of larvae and pupal diapause make this insect difficult to control using conventional insecticides [20, 21]. Some methods have been developed to monitor population outbreaks of *B*. *minax*, and the transcriptome of *B*. *minax* has been determined [22]. Olfactory proteins that are crucial in allowing the insect to locate potential oviposition substrates (citrus fruit), and food lures to attract adult *B*. *minax* have been developed [23, 24]. Host plant volatiles which synergize the response to sex pheromones in the orange have attracted attention [25]. However, the olfactory responses of *B*. *minax* to different host plants and the genes involved are yet to be elucidated.

In our study, we identified functional olfactory molecules in *B*. *minax* and evaluated the responses of the fly to its specific attractant volatile D-limonene. RNA interference technique revealed the predominance of *BminCSP* and *BminOBP21* genes in olfactory and non-olfactory tissues, specifically in the antennae of *B*. *minax*. Compared with GFP-injected *B*. *minax* (control), RNAi-treated *B*. *minax* had significantly lower electrophysiological responses to D-limonene. Our data add a unique understanding of the molecular olfactory responses of *B*. *minax* that will facilitate the development of attractants for an effective biological control approach of *B*. *minax*.

## Methods

### Ethical statement

*Bactrocera minax* is a pest insect which does not require any permission for their manipulation and handling. The study was approved by the College of Plant Science and Technology, Huazhong Agricultural University.

### Insect rearing and maintenance

The third instar larvae of *B*. *minax* were retrieved from infested citrus fruits planted in San Douping county, Hubei province, China. Adults were kept in cubical cages (50cm x 50cm x 50cm) and fed with sucrose and brewer’s yeast at 28°C, under relative humidity of 70–80% with light -dark ratio of 14 h: 10 h.

### Transcriptome analysis and functional annotation

*B*. *minax* heads were dissected from newly emerged females, sexually mature males, and sexually mature females, respectively. The heads were gently separated using sterilized forceps under a stereomicroscope, washed twice in DEPC-ethanol 70% and used immediately. The total RNA was isolated using RNAiso plus reagent (TaKaRa Biotechnology, China). Transcriptome analyses were performed according to previously published methods [26].

The difference in gene expression at different stages was compared using FPKM of genes from all samples of the transcriptome. The Blast2GO program was adopted for functional annotation of the genes [27]. The open reading frame (ORF) of the identified unigene was predicted by ORF Finder and verified on the basis of protein BLAST results [28]. The signal peptides of OBPs and CSPs were predicted by SignalP 4.0. The transmembrane domain (TMD) of the identified OR was evaluated by TMHMM server v. 2.0 [29].

### Phylogenetic analyses

Based on the amino acid sequences of candidate olfaction genes and collected olfaction genes, the phylogenetic tree was established in MEGA 7.0 software. Clustal W was performed to align the amino acid sequences. A bootstrap procedure was carried out to assess node support [30].

### qRT-PCR-based analysis of candidate olfactory gene expression

qRT-PCR analysis was performed to evaluate the expression profiles of the putative olfactory genes from different samples[15]. Total RNA was extracted according to the method mentioned above. cDNA was synthesized using a first strand cDNA Synthesis Kit. 10 μl of the PCR master mix consisted of 5 μl of TB Green Premix Ex TaqII, 0.2 μl of ROX Reference Dye, 0.6 μl of cDNA templates, 0.4 μl of each primer, and 3.4 μl of double-distilled water. Primers are described in S4 Table. Three biological samples were analyzed for each experiment. The expression level of olfactory gene was quantified and calculated using the 2^−ΔΔCT^ method with the *Bmtubulin* gene as control [31].

### RNA interference and electrophysiological recordings

Full-length *BminCSP* and *BmOBP21* dsRNA was synthesized through *in vitro* transcription and purified using RNeasy MinElute Cleanup Kit. About 100 nl of dsRNA was injected into sexually mature female *B*. *minax* with a micro Injector™ System MINJ-1. Two lines of injected flies were generated, namely the dsRNA-injected and dsGFP-injected ones. Individual female head was dissected 2 days after injection. RNA was extracted from each head and qRT-PCR was conducted using the same methods as earlier described, three biological samples and three technical repeats were analyzed for each treatment [32]. Primers used in RNAi for PCR and qRT-PCR are described in the supporting information (S5 and S6 Tables).

An antennae of an adult *B*. *minax* female was excised and mounted on a Syntech EAG platform. One metal conductive electrode was used for reference while the other was used as recording electrode [33]. The antennal preparation was bathed in a humidified air stream flowing at 20 ml/s. *B*. *minax* is sensitive to D-limonene [25]. D-limonene (99%, Sigma-Aldrich, USA) was dissolved in normal hexane (99%), resulting in a stock solution of 1 μg/μl. 10 μl of the stimulus was loaded onto a filter paper strip, and then introduced in 1 ml of polypropylene syringe. Solvent blanks of equal volume served as controls. The order in which antennae receive odor stimulation was solvent blank, then EAG (std1), followed by stimuli (EAG(A), then another solvent blank, and finally EAG (std2). rEAG is the relative EAG response. Each treatment contained a minimum of five replicates.

$$rEAG=\frac{2\mathrm{EAG}\left(A\right)}{EAG\left(std1\right)+EAG\left(std2\right)}$$

### Statistical analysis

One-way ANOVA was performed to analyze the gene expression in SPSS 22.0 software. The relative gene expression level between dsRNA treatment and control was evaluated by *t*-tests at α = 0.05. In contrast, the rEAG between dsRNA treatment and control was evaluated by *t*-tests at α = 0.05. The difference was statistically significant when *P* < 0.05.

## Results

### Putative chemosensory proteins identification

**Fig 1:**
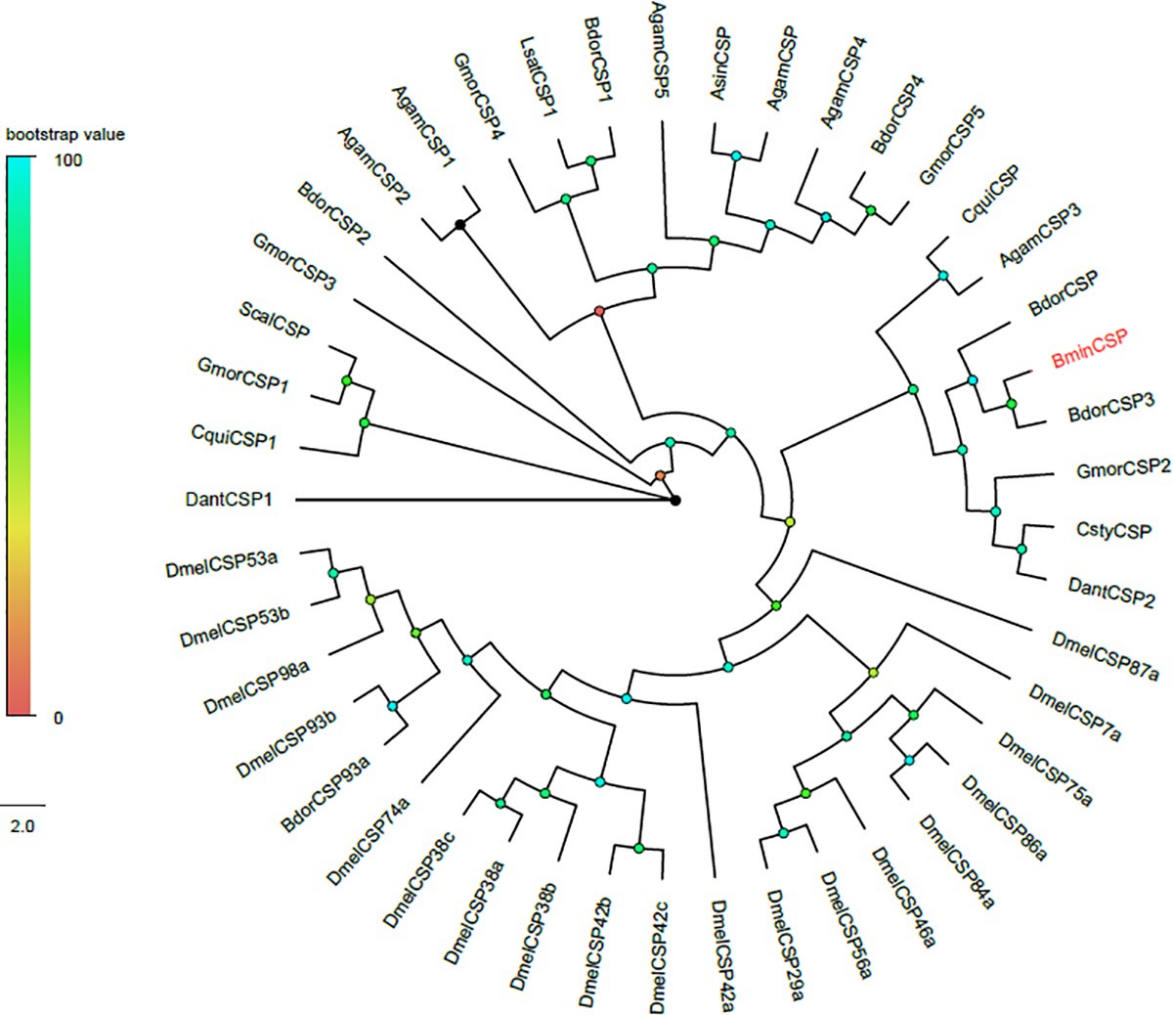
Phylogenetic tree of candidate *BminCSP* with other Dipteran CSP sequences. Dmel: *Drosophila melanogaster;* Bdor: *Bactrocera dorsalis;* Cqui: *Culex quinquefasciatus;* Csty: *Calliphora stygia;* Gmor: *Glossina morsitans morsitans;* Dant: *Delia antiqua*; Scal: *Stomoxys calcitrans* Asin: *Anopheles sinensis;* Agam: *Anopheles gambiae;* Lsat: *Liriomyza sativae*.

As shown in S1 Table, a candidate CSP was identified and predicted to have a full sequence without signal peptide. From the Neighbor-Joining tree, it could be found that the sequences were clustered with orthologous gene, which could be easily identified, as shown in Fig 1. The unigene *BminCSP* was predicted to have the same function with *BdorCSP3* in terms of feeding and oviposition [34].

### Putative odorant-binding proteins identification

**Fig 2:**
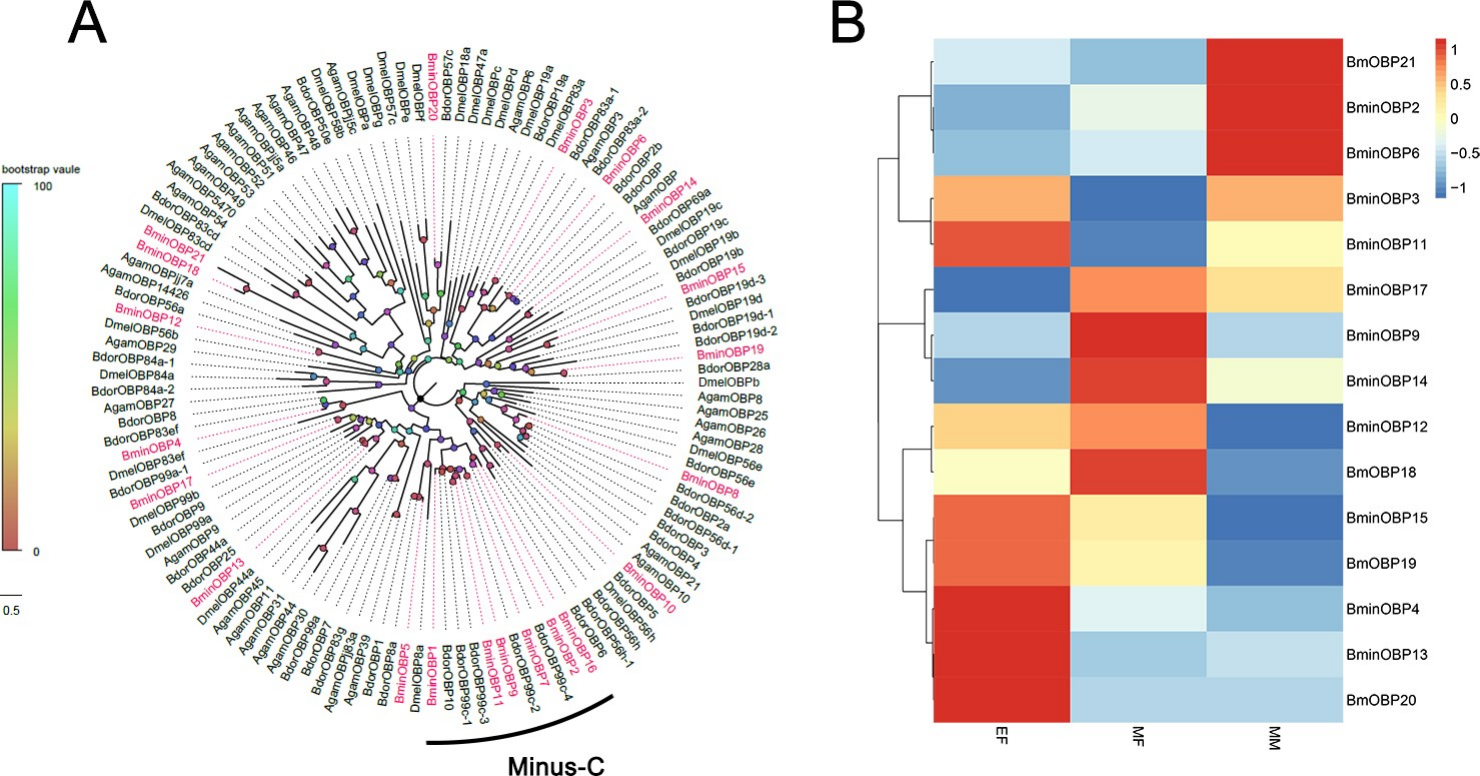
Phylogenetic tree of candidate *BminOBPs* with known Dipteran OBP sequences and the candidate *BminOBP* genes expression pattern in newly emerged females (EF), sexually mature females (MF), and sexually mature males (MM). Dmel: *D melanogaster;* Bdor: *B dorsalis;* Agam: *A gambiae*.

By aligning the 21 candidate OBPs to each other, they were organized into different classes according to the number of cysteine motifs present in each transcript and a phylogenetic was constructed, as shown in Fig 2A. All putative OBPs were similar to known OBPs from other Dipteran species. The identified OBP genes clustered in different subgroups and encoded a variety of proteins. A few genes that clustered together with their counterparts from *B*. *dorsalis* were identified and named as *BminOBP1*, *BminOBP2*, *BminOBP7*, *BminOBP9*, *BminOBP11*, and *BminOBP16*, respectively (S1 Table). According to the heatmap, *BminOBP2*, *BminOBP6* were highly expressed in mature males (MM) while *BminOBP9*, *BminOBP14*, *BminOBP12* were highly expressed in mature females (MF), and *BminOBP4*, *BminOBP11*, *BminOBP13* were highly expressed in newly emerged females (EF) (Fig 2B).

### Identification of candidate olfactory receptor proteins

**Fig 3:**
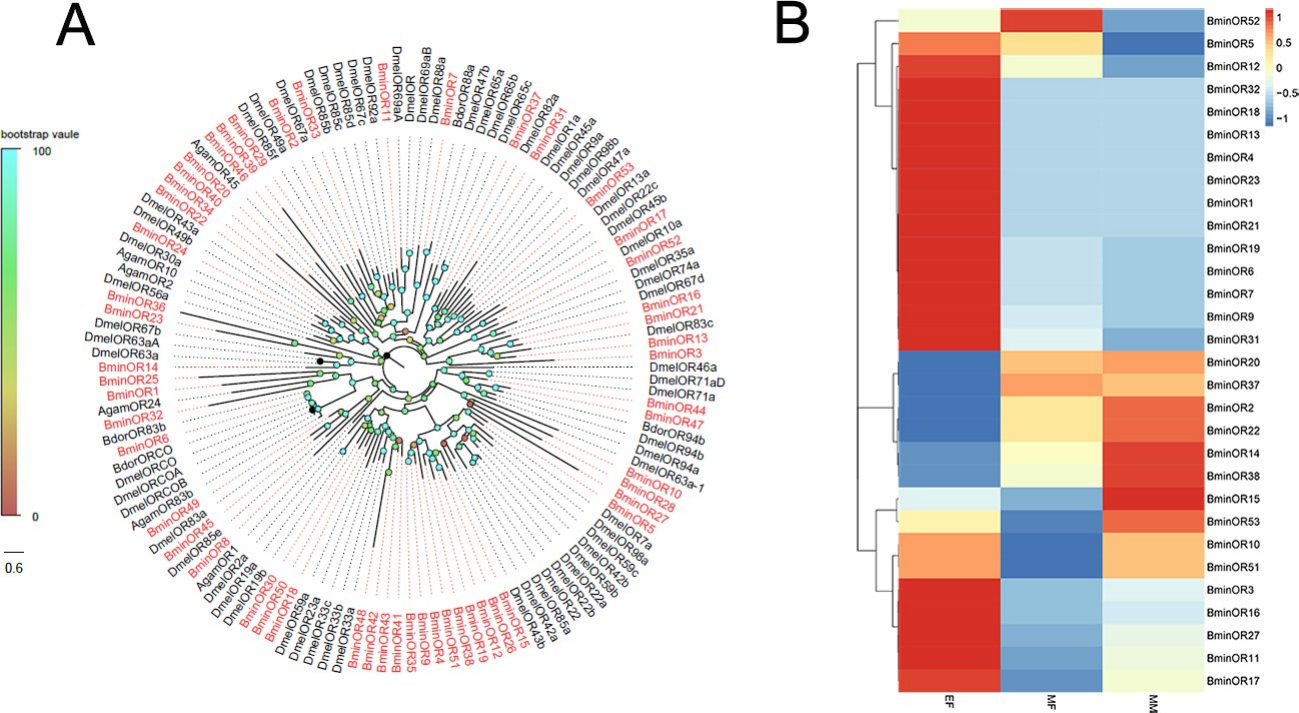
Phylogenetic tree of candidate *BminORs* with known Dipteran OR sequences and the candidate *BminOR* genes expression pattern in EF, MF and MM. Dmel: *D melanogaster;* Bdor: *B dorsalis;* Agam: *A gambiae*.

Transcripts encoding 53 putative olfactory receptors (ORs) were identified. Among them, five were full-length genes encoding proteins of more than 399 amino acids. The unigene reference, length, and BLASTx best hit of all OR are shown in S2 Table. The majority of OR candidate genes clustered with at least one orthologous gene, forming multiple lineages (Fig 3A). Moreover, *BminOR9*, *BminOR16*, *BminOR19*, *BminOR21*, *BminOR23*, *BminOR27*, *BminOR31* and *BminOR32* genes were highly expressed in newly emerged flies (EF), while *BminOR38* gene was highly expressed in sexually mature males (MM) (Fig 3B).

### Identification of candidate ionotropic receptors

**Fig 4:**
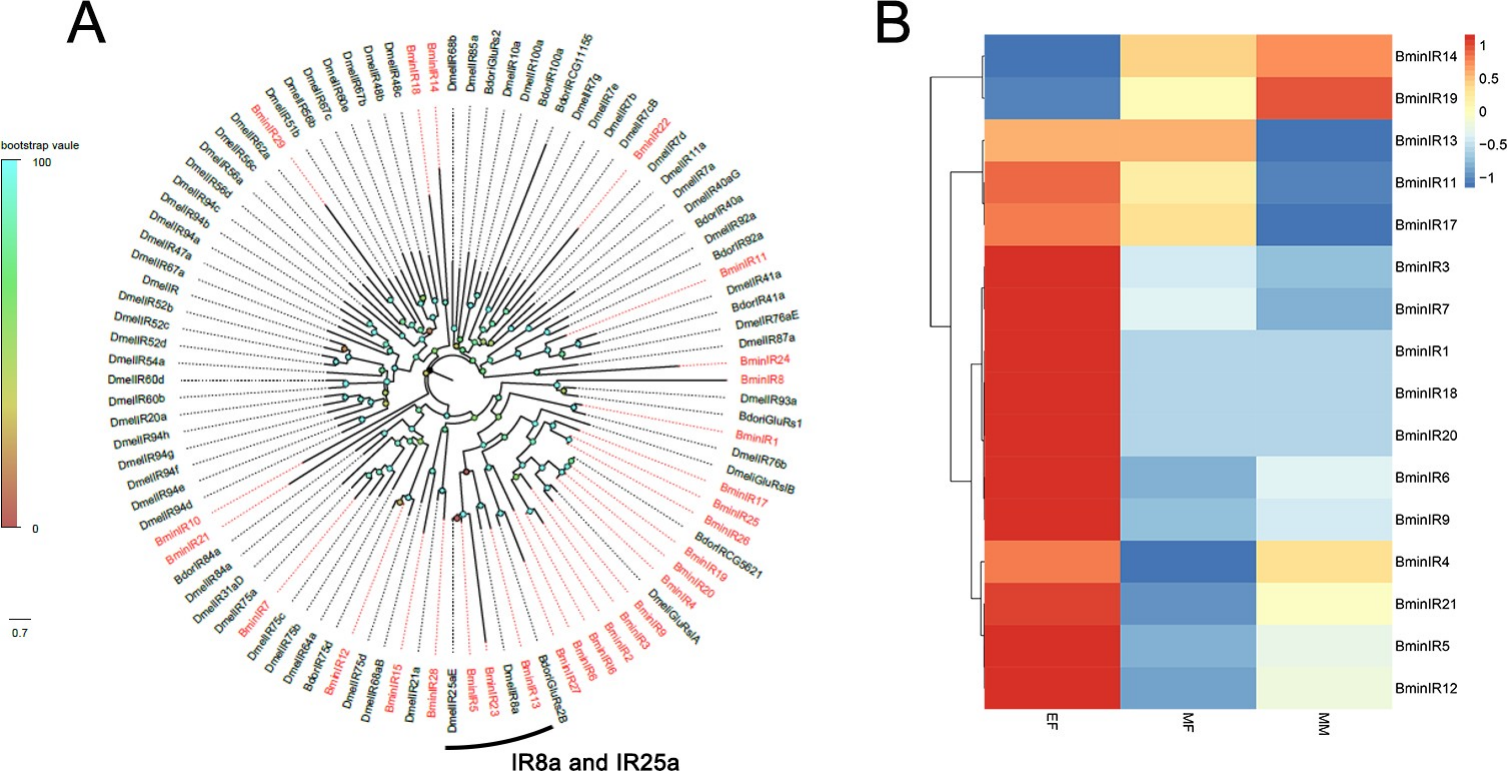
Phylogenetic tree of candidate *BminIRs* with known Dipteran IR sequences and the candidate *BminIR* genes expression pattern in EF, MF and MM. Dmel: *D melanogaster;* Bdor: *B dorsalis*.

A total of 29 candidate IR sequences were identified in *B*. *minax* transcriptomic analyses, of which, 5 IRs had complete open reading frames (ORF), whereas the others were represented as partial ORF. An unrooted phylogenetic tree was established to reveal the relationship among the IRs from *B*. *minax* and other Dipteran species (Fig 4A). The name, unigene reference, length, and best BLASTx hit of all 29 IRs are shown in S3 Table. The heatmap revealed predominant expressions of *BminIR1*, *BminIR11*, *BminIR12* in EF, while *BminIR21* was highly expressed in MM and MF (Fig 4B).

### Identification of candidate SNMPs

Four candidate SNMPs were identified from the *B*. *minax* transcriptome, including. *BminSNMP1a*, *BminSNMP1b*, *BminSNMP1c*, and *BminSNMP2a*. The protein sequences of the SNMPs are shown in S1 Text.

### Expression pattern of candidate olfactory gene

**Fig 5:**
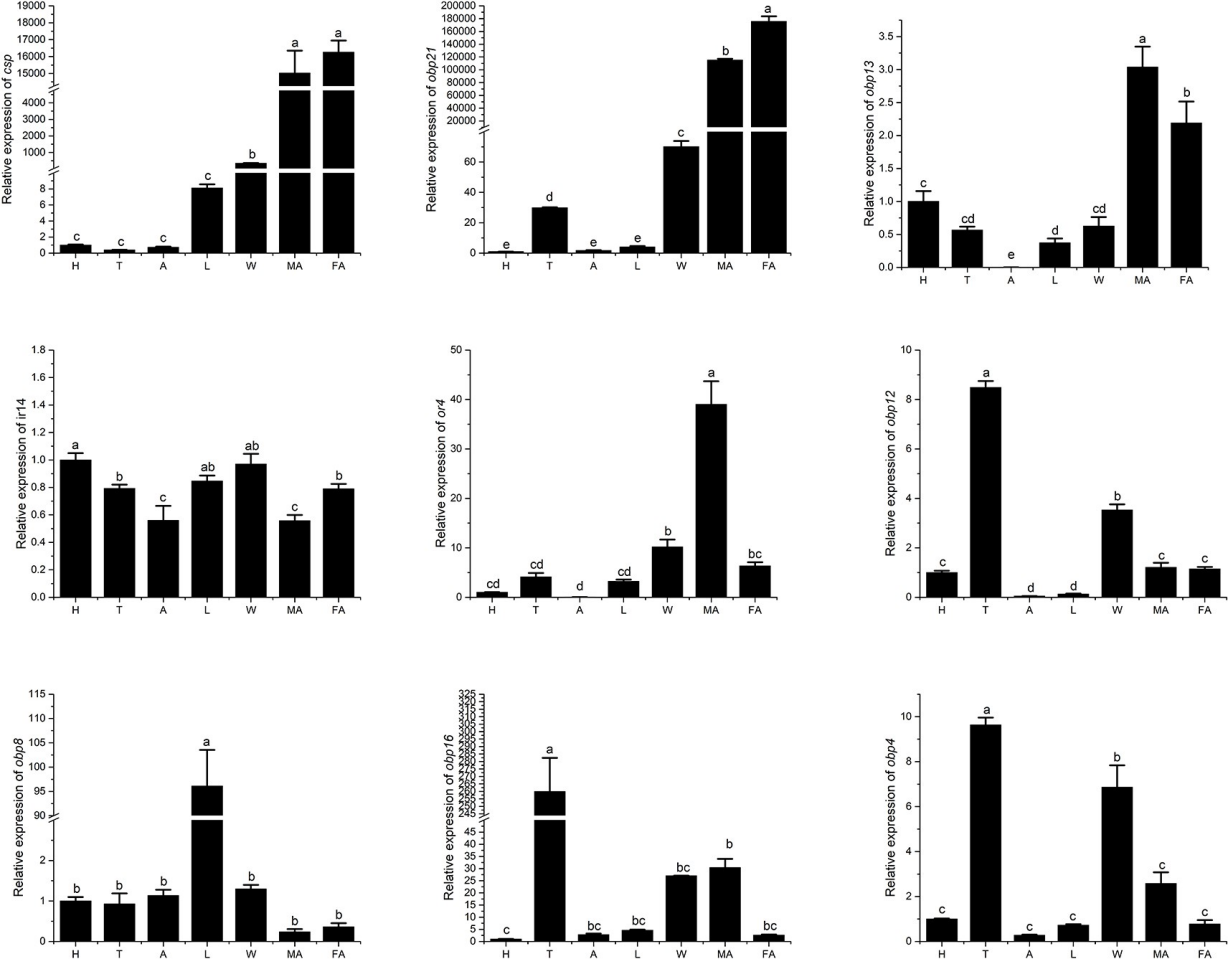
Tissue- and sex-specific expression patterns of candidate *B*. *minax* olfaction genes. The X-axis represents the different tissues of *B*. *minax*. FA: female antennae; MA: male antennae; H: head (without antennae); T: thorax; A: abdomen; L: legs; W: wings. Error bars represent the standard error of the measurement.

**Fig 6:**
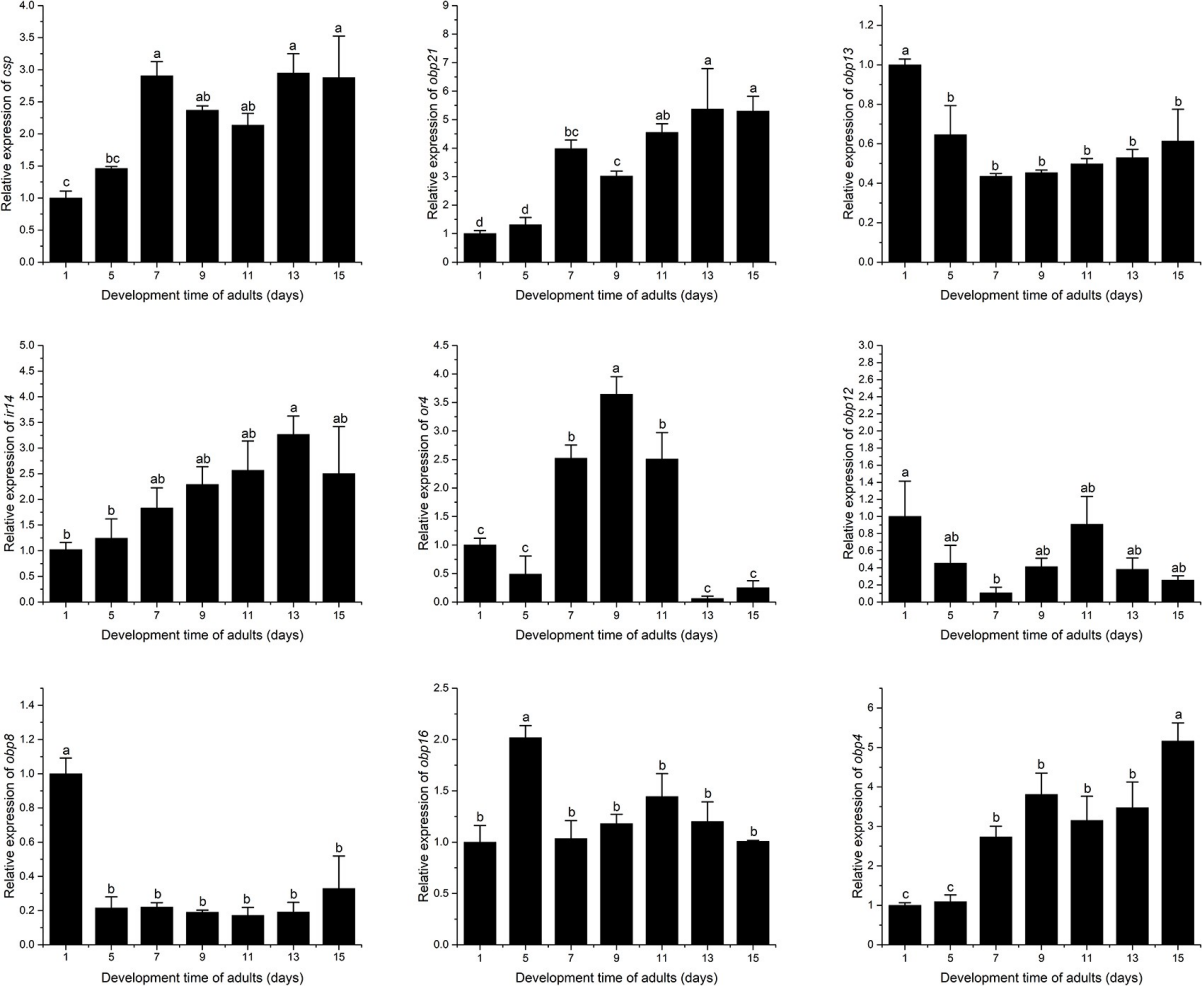
Time expression patterns of candidate *B*. *minax* olfaction genes. Error bars represent 1 SE.

qRT-PCR was carried out to investigate the expression pattern of the candidate olfactory genes in male antennae, female antennae, head, thorax, abdomen, leg, and wing. As shown in Fig 5, all examined genes could be detected in *B*. *minax* antennae, but only some of the genes could be identified in other parts of the body (Fig 5). It was worth noting that *BminCSP*, *BminOBP13*, and *BminOBP21* were highly expressed in antennae of both male and female, *BminOBP8* was highly expressed only in the leg, while *BminOBP16*, *BminOBP4*, and *BminOBP12* were highly expressed in the thorax and *BminOR4* was highly expressed in antennae of males only. Moreover, the expressions of *BminCSP*, *BminOBP4*, *BminOBP21*, *BminIR14* genes reached their peaks at the 13^th^ day when *B*. *minax* was becoming sexually mature, indicating their potential involvement in ovary development and oviposition (Fig 6).

### Electrophysiological recordings

**Fig 7:**
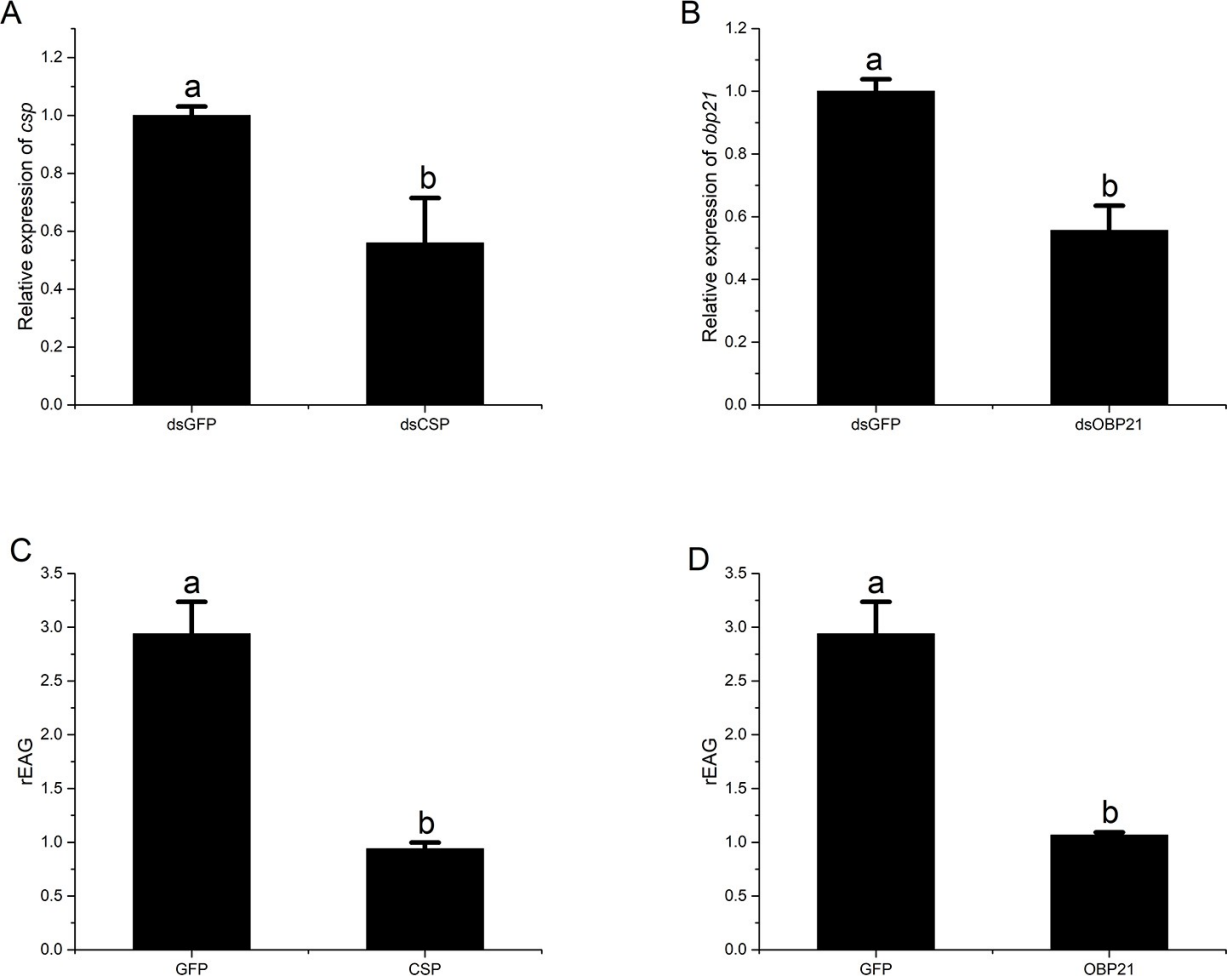
EAG response of *B*. *minax* antennae to D-limonene for the sexually mature females. A: qRT-PCR result of *BminCSP* gene silencing; B: qRT-PCR result of *BminOBP21* gene silencing; C, D: Relative EAG response of GFP- and RNAi-treated female to D-limonene.

The qRT-PCR analysis was performed to examine mRNA levels of *BminCSP* and *BminOBP21* in the antennae of dsRNA-injected insects and GFP-injected insects (control). Compared with GFP-injected insects, *BminCSP* and *BminOBP21* transcript levels in dsRNA injected *B*. *minax* were significantly reduced, as shown in Fig 7. Through investigating EAG response to attractant of control and RNAi-treated *B*. *minax* females, it could be known that silencing *BminCSP* and *BminOBP21* genes significantly influenced antennal response to D-limonene (Fig 7).

## Discussion

OBPs and CSPs are highly expressed in the sensillum lymph and involved in the first critical step in odorant detection [13]. It has been reported that OBPs and CSPs probably carry semiochemicals affecting the behavior [34].

*B*. *minax*, is a critical phytophagous pest, which has been largely spread across China in recent years and gradually become a significant threat to worldwide citrus industry [21].The mechanism underlying the chemical communication in *B*. *minax* has rarely been researched [25]. Therefore, it is of great importance to determine the genes that are responsible for semiochemical perception [6, 16]. Candidate olfactory genes have been identified through transcriptomic analyses and annotation. In an attempt to unravel the molecular basis of olfaction in *B*. *minax*, we studied the whole transcriptomes from the head and antennae of this pest, and then, we determined the number of genes involved in olfactory processes.

A total of 108 putative olfactory genes (1 CSP, 21 OBPs, 53 ORs, 29 IRs, and 4 SNMPs) were identified from the transcriptome of *B*. *minax*. This number is somewhat lower than that detected in *B*. *dorsalis*, a polyphagous insect pest with a diverse host range. For *B*. *dorsalis*, it consists of 155 olfactory genes (3 CSPs, 35 OBPs, 74 ORs, 40 IRs, and 3 SNMPs) at the transcriptome level (our unpublished data). This could be an indication that the development of olfactory perception in fruit fly depends on the host plant range of the fly, either monophagous (eating of one host plant), oligophagous (feeding on a few specific hosts) or polyphagous (feeding on a broad spectrum of host fruits), since *B*. *minax* is an oligophgaous insect while *B*. *dorsalis* is a polyphagous one. Another possibility is that *B*. *minax* may use visual as well as olfactory signals to find and locate the suitable host plants and fruits [26].

Transcriptome analysis has been carried out to identify the differentially expressed genes in a specific tissue of insects. The BLAST analysis of the OBP and CSP proteins identified in *B*. *minax* allowed us to characterize the sequences [17]. In this work, the transcriptome analysis revealed the identification of 1 CSP and 1 OBP (named as *BminCSP* and *BminOBP21*) which were highly expressed in antennae of both male and female *B*. *minax*. The injection of dsRNA targeting *BminCSP* and *BminOBP21* genes significantly affected the antennal responses to D-limonene, which is a putative attractant that normally activates antennal responsiveness for oviposition or host location. Moreover, *BminOBP8* gene was highly expressed in the legs, which may indicate its involvement in *B*. *minax* locomotion and host location.

The elementary functional and structural characteristics of insect olfactory receptor have remained unknown [10, 35]. In this study, 53 odorant receptors (ORs) candidate genes were identified. Male-specific ORs play a role in pheromone detection, while female-specific ORs are expected to feature in oviposition-related odorant detection [36]. For most species, only one obligate co-receptor (Orco) is expressed, which is a distinct complement of ORs [37].

The detection of one Orco gene (*BminOR6*) in our study could indicate the extent of its involvement in the olfaction of *B*. *minax*. A similar result was obtained in the vinegar fly *Drosophila melanogaster*, in which the odorant receptor gene was highly involved in its olfaction system. Although the molecular mechanisms of olfaction driven by *BminOR6* gene in *B*. *minax* require further study, our results could constitute a starting point for implementing novel control strategies by targeting the olfaction properties of the fly. The significant expression of *BminIR21* (ionotropic receptor candidate gene) in mature stages of the fly is possibly linked to the host maintenance and foraging ability. A recent study showed that a part of IR subtypes is involved in the detection of food-derived odors, while the other part is tuned to polyamines [12]. The two SNMPs of *B*. *minax* have been published [38]. *BminSNMP1a* identified in our study is nearly the same with *BminSNMP1* in published paper, while *BminSNMP2a* identified in our study is an orthologous gene with *BminSNMP2*.

Olfaction plays a key role in locating food, sexual partners, and oviposition sites [6, 39]. At present, the techniques for studying gene function include RNAi [40] and CRISPR/Cas9 technologies [41]. Some olfactory genes functions have been identified in insects by RNAi [42]. In *B*. *dorsalis*, silencing OBP genes reduced the fecundity of females [40]. Currently, reverse chemical ecology, such as linking insect olfactory proteins to their respective pheromone and plant kairomones is the key and could provide a novel method for researching sophisticated mechanisms of chemosensory perception in insects [43].

Based on the present work, we are tentatively exploring the underlying molecular mechanisms of olfaction and chemoreception in *B*. *minax* to better understand how those candidate genes could be adequately manipulated for implementing effective management strategies of *B*. *minax* in the near future.

## Conclusions

Overall, we firstly identified a total of 108 new olfactory genes in *B*. *minax*, including 1 CSP, 21 OBPs, 53 ORs, 29 IRs, and 4 SNMPs. This provides theoretical basis for investigating the mechanisms of olfaction in *B*. *minax*. In this study, we established a phylogenetic tree of olfactory genes. The results indicated that most of olfactory genes were expressed in the chemosensory organs while some genes showed antenna-biased expression. Moreover, the knock down of *BminCSP* and *BminOBP21* genes affected antennal responses to D-limonene, a putative specific attractant. This study provides theoretical basis for researches on olfactory system of *B*. *minax*, and the variety of genes identified could constitute potential targets for genetic-based pest management against this notorious pest and other related pests.

## Supporting information

S1 Table Unigenes of candidate chemosensory proteins and odorant binding proteins. (DOCX)

S2 Table Unigenes of candidate olfactory receptors. (DOCX)

S3 Table Unigenes of candidate ionotropic receptors. (DOCX)

S4 Table Primers used in the qRT-PCR experiments for expression patterns of candidate olfactory genes. (DOCX)

S5 Table Primers used in RNAi for PCR. (DOCX)

S6 Table Primers used in RNAi for qRT-PCR. (DOCX)

S1 Text Fasta format of the protein sequences of OBPs, CSPs, ORs, IRs and SNMPs identified in this study. (DOCX)
