# Supplementary material for: Identification of olfactory genes and functional analysis of BminCSP and BminOBP21 in Bactrocera minax
Source: PLoS One. 2019 Sep 11;14(9):e0222193. doi: 10.1371/journal.pone.0222193 (PMC6739056; doi:10.1371/journal.pone.0222193)
Supplement: S1 Table — (DOCX) [file pone.0222193.s001.docx]

**S1 Table** Unigenes of candidate chemosensory proteins and odorant binding proteins

| Gene name | Length  (nt) | ORF (aa) | Unigene reference | Status | Signal  Peptide | Evalue | BLASTx best hit |
| --- | --- | --- | --- | --- | --- | --- | --- |
| *BminCSP* | 998 | 169 | CL9660.Contig2_All | Complete ORF | N | 1E-93 | gb\|ACB56576.1\|chemosensory protein [Bactrocera dorsalis] |
| *BminOBP1* | 1868 | 172 | CL5478.Contig1_All | Complete ORF | N | 2E-88 | ref\|XP_011184698.1\|general odorant-binding protein 99a-like [Bactrocera cucurbitae] |
| *BminOBP2* | 693 | 152 | CL5869.Contig1_All | Complete ORF | Y | 2E-100 | gb\|AGC82130.1\|odorant-binding protein 1 [Bactrocera dorsalis] |
| *BminOBP3* | 4275 | 165 | CL6829.Contig4_All | Complete ORF | Y | 7E-114 | ref\|XP_011212469.1\|pheromone-binding protein-related protein 6 [Bactrocera dorsalis] |
| *BminOBP4* | 1885 | 289 | CL7376.Contig1_All | Complete ORF | N | 0 | gb\|AKI29018.1\| odorant binding protein 83ef [Bactrocera dorsalis] |
| *BminOBP5* | 1473 | 162 | CL8140.Contig2_All | Complete ORF | Y | 3E-105 | gb\|AGS08183.1\| odorant binding protein 1 [Bactrocera dorsalis] |
| *BminOBP6* | 762 | 154 | CL9536.Contig1_All | 5'lost | Y | 3E-105 | gb\|AKM45844.1\| odorant-binding protein 2 [Bactrocera dorsalis] |
| *BminOBP7* | 678 | 154 | Unigene6877_All | Complete ORF | Y | 1E-100 | ref\|XP_011210426.1\|general odorant-binding protein 99a-like [Bactrocera dorsalis] |
| *BminOBP8* | 815 | 168 | Unigene15302_All | Complete ORF | N | 7E-68 | ref\|XP_011180835.1\|general odorant-binding protein 56d-like [Bactrocera dorsalis] |
| *BminOBP9* | 413 | 136 | Unigene17830_All | Complete ORF | Y | 8E-73 | ref\|XP_011210445.1\|general odorant-binding protein 99a-like [Bactrocera dorsalis] |
| *BminOBP10* | 575 | 145 | Unigene19898_All | Complete ORF | N | 8E-63 | gb\|AGS08187.1\|odorant binding protein 5 [Bactrocera dorsalis] |
| *BminOBP11* | 499 | 161 | Unigene24992_All | Complete ORF | Y | 7E-96 | gb\|AKI29025.1\|odorant binding protein 99c-3 [Bactrocera dorsalis] |
| *BminOBP12* | 675 | 177 | CL4030.Contig1_All | Complete ORF | N | 2E-10 | AKI29008.1 odorant binding protein 56a [Bactrocera dorsalis] |
| *BminOBP13* | 2724 | 161 | CL5860.Contig1_All | Complete ORF | N | 1E-98 | XP_011182910.1 general odorant-binding protein 99a [Bactrocera cucurbitae] |
| *BminOBP14* | 3496 | 567 | CL6119.Contig2_All | Complete ORF | Y | 1E-82 | XP_014088831.1 general odorant-binding protein 69a [Bactrocera oleae] |
| *BminOBP15* | 1309 | 150 | CL6511.Contig5_All | 5'lost | N | 2E-79 | AKM45840.1 odorant-binding protein 20 [Bactrocera dorsalis] |
| *BminOBP16* | 2196 | 180 | CL6787.Contig3_All | 5'lost | N | 6E-63 | XP_014090663.1 general odorant-binding protein 99b-like [Bactrocera oleae] |
| *BminOBP17* | 753 | 153 | CL10197.Contig2_All | Complete ORF | Y | 3E-109 | AKM45835.1 odorant-binding protein 15, partial [Bactrocera dorsalis] |
| *BminOBP18* | 576 | 98 | CL10347.Contig1_All | 5'lost | N | 1E-57 | XP_011210114.1 putative odorant-binding protein A5 [Bactrocera dorsalis] |
| *BminOBP19* | 680 | 176 | CL11209.Contig1_All | Complete ORF | N | 2E-102 | XP_011198820.1 general odorant-binding protein 28a-like [Bactrocera dorsalis] |
| *BminOBP20* | 863 | 205 | Unigene25090_All | Complete ORF | N | 4E-103 | XP_014091324.1 general odorant-binding protein 57c [Bactrocera oleae] |
| *BminOBP21* | 1035 | 220 | CL717.Contig1_All | Complete ORF | N | 7E-136 | XP_011210072.1 putative odorant-binding protein A5 [Bactrocera dorsalis] |
