# Supplementary material for: Identification of olfactory genes and functional analysis of BminCSP and BminOBP21 in Bactrocera minax
Source: PLoS One. 2019 Sep 11;14(9):e0222193. doi: 10.1371/journal.pone.0222193 (PMC6739056; doi:10.1371/journal.pone.0222193)
Supplement: S2 Table — (DOCX) [file pone.0222193.s002.docx]

**S2 Table** Unigenes of candidate olfactory receptors

| Gene name | Length  (nt) | ORF (aa) | Unigene reference | Status | TMD  (No.) | Evalue | BLASTx best hit |
| --- | --- | --- | --- | --- | --- | --- | --- |
| *BminOR1* | 1591 | 467 | CL273.Contig1_All | Complete ORF | 6 | 0 | ref\|XP_011204023.1\|gustatory and odorant receptor 21a-like [Bactrocera dorsalis] |
| *BminOR2* | 331 | 110 | CL653.Contig1_All | 5'lost | 2 | 6E-72 | ref\|XP_011200401.1\|odorant receptor 67c-like [Bactrocera dorsalis] |
| *BminOR3* | 739 | 230 | CL1678.Contig2_All | 5'lost | 3 | 2E-150 | ref\|XP_014095104.1\|gustatory and odorant receptor 63a [Bactrocera oleae] |
| *BminOR4* | 519 | 126 | CL2339.Contig1_All | 5'lost | 3 | 2E-63 | ref\|XP_011189274.1\|odorant receptor 7a-like [Bactrocera cucurbitae] |
| *BminOR5* | 1006 | 296 | CL3657.Contig1_All | 5'lost | 4 | 0 | ref\|XP_011209572.1\|odorant receptor 7a-like [Bactrocera dorsalis] |
| *BminOR6* | 2928 | 480 | CL4023.Contig2_All | Complete ORF | 7 | 0 | ref\|XP_011203778.1\|odorant receptor coreceptor [Bactrocera dorsalis] |
| *BminOR7* | 579 | 192 | CL5015.Contig2_All | 5'lost | 1 | 6E-123 | ref\|XP_011183038.1\|odorant receptor 88a [Bactrocera cucurbitae] |
| *BminOR8* | 246 | 81 | CL5023.Contig2_All | 5'lost | 0 | 8E-51 | ref\|XP_014101212.1\|gustatory and odorant receptor 21a-like, partial [Bactrocera oleae] |
| *BminOR9* | 1361 | 399 | CL5452.Contig1_All | Complete ORF | 4 | 0 | ref\|XP_011198720.1\|odorant receptor 7a-like [Bactrocera dorsalis] |
| *BminOR10* | 449 | 149 | CL5620.Contig2_All | 5'lost | 1 | 3E-104 | ref\|XP_011204429.1\|odorant receptor 13a [Bactrocera dorsalis] |
| *BminOR11* | 1541 | 416 | L6165.Contig2_All | Complete ORF | 6 | 0 | ref\|XP_011209369.1\|putative odorant receptor 69a, isoform B [Bactrocera dorsalis] |
| *BminOR12* | 1064 | 327 | CL6814.Contig1_All | 5'lost | 3 | 0 | ref\|XP_014086206.1\|odorant receptor 43b-like [Bactrocera oleae] |
| *BminOR13* | 417 | 139 | CL6874.Contig2_All | 5'lost | 2 | 2E-83 | ref\|XP_014091805.1\| odorant receptor 67d-like [Bactrocera oleae] |
| *BminOR14* | 1178 | 320 | CL7465.Contig2_All | 5'lost | 5 | 0 | ref\|XP_011201815.1\|odorant receptor 63a-like [Bactrocera dorsalis] |
| *BminOR15* | 1576 | 409 | CL7824.Contig3_All | Complete ORF | 4 | 0 | gb\|AKI29030.1\|odorant receptor 7a-3 [Bactrocera dorsalis] |
| *BminOR16* | 846 | 271 | CL8040.Contig1_All | 5'lost | 4 | 7E-100 | ref\|XP_014097995.1\|odorant receptor 67d-like [Bactrocera oleae] |
| *BminOR17* | 1078 | 165 | CL8583.Contig1_All | 5'lost | 3 | 4E-82 | ref\|XP_014091911.1\|odorant receptor 10a [Bactrocera oleae] |
| *BminOR18* | 387 | 128 | CL8872.Contig1_All | 5'lost | 2 | 1E-85 | ref\|XP_011198390.1\|odorant receptor 2a-like [Bactrocera dorsalis] |
| *BminOR19* | 1742 | 439 | CL9931.Contig2_All | 5'lost | 5 | 9E-180 | ref\|XP_011178079.1\|odorant receptor 7a-like [Bactrocera cucurbitae] |
| *BminOR20* | 1439 | 271 | CL10543.Contig1_All | 5'lost | 5 | 1E-83 | ref\|XP_014100035.1\|odorant receptor 30a-like [Bactrocera oleae] |
| *BminOR21* | 925 | 269 | CL11237.Contig2_All | 5'lost | 4 | 3E-61 | ref\|XP_004533438.1\| odorant receptor 67d-like [Ceratitis capitata] |
| *BminOR22* | 305 | 101 | CL11449.Contig2_All | 5'lost | 2 | 5E-60 | ref\|XP_011178893.1\|odorant receptor 43a [Bactrocera cucurbitae] |
| *BminOR23* | 481 | 157 | Unigene1168_All | 5'lost | 3 | 1E-105 | gb\|AKI29034.1\|odorant receptor 35a [Bactrocera dorsalis] |
| *BminOR24* | 626 | 208 | Unigene1444_All | 5'lost | 4 | 1E-150 | gb\|AKI29039.1\|odorant receptor 49b-1 [Bactrocera dorsalis] |
| *BminOR25* | 427 | 141 | Unigene7171_All | 5'lost | 3 | 4E-55 | ref\|XP_011194820.1\|odorant receptor Or2-like [Bactrocera cucurbitae] |
| *BminOR26* | 453 | 133 | Unigene8286_All | 5'lost | 2 | 2E-57 | gb\|AKI29037.1\|odorant receptor 43b [Bactrocera dorsalis] |
| *BminOR27* | 486 | 127 | Unigene8962_All | 5'lost | 2 | 2E-82 | ref\|XP_011208732.1\|odorant receptor 82a [Bactrocera dorsalis] |
| *BminOR28* | 332 | 110 | Unigene9637_All | 5'lost | 2 | 1E-70 | ref\|XP_011208819.1\|putative odorant receptor 92a [Bactrocera dorsalis] |
| *BminOR29* | 333 | 110 | Unigene9765_All | 5'lost | 2 | 9E-68 | ref\|XP_011208819.1\|putative odorant receptor 92a [Bactrocera dorsalis] |
| *BminOR30* | 257 | 85 | Unigene12111_All | 5'lost | 1 | 1E-51 | ref\|XP_011187627.1\|odorant receptor 2a-like [Bactrocera cucurbitae] |
| *BminOR31* | 717 | 239 | Unigene13284_All | 5'lost | 2 | 3E-166 | ref\|XP_011212447.1\|odorant receptor 45a-like [Bactrocera dorsalis] |
| *BminOR32* | 372 | 123 | Unigene13695_All | 5'lost | 2 | 6E-10 | ref\|XP_011203704.1\|odorant receptor 67d-like [Bactrocera dorsalis] |
| *BminOR33* | 371 | 123 | Unigene14022_All | 5'lost | 2 | 3E-58 | ref\|XP_011209576.1\|odorant receptor 85c-like [Bactrocera dorsalis] |
| *BminOR34* | 608 | 161 | Unigene14034_All | 5'lost | 2 | 5E-80 | ref\|XP_014097486.1\|odorant receptor Or2-like [Bactrocera oleae] |
| *BminOR35* | 349 | 115 | Unigene14112_All | 5'lost | 2 | 4E-71 | ref\|XP_011198720.1\|odorant receptor 7a-like [Bactrocera dorsalis] |
| *BminOR36* | 256 | 84 | Unigene14281_All | 5'lost | 2 | 9E-53 | ref\|XP_011200401.1\|odorant receptor 67c-like [Bactrocera dorsalis] |
| *BminOR37* | 1071 | 262 | Unigene14938_All | 5'lost | 4 | 0 | ref\|XP_011208732.1\|odorant receptor 82a [Bactrocera dorsalis] |
| *BminOR38* | 631 | 174 | Unigene20612_All | 5'lost | 1 | 8E-81 | ref\|XP_011187808.1\|odorant receptor 7a-like [Bactrocera cucurbitae] |
| *BminOR39* | 422 | 137 | Unigene24657_All | 5'lost | 2 | 1E-37 | ref\|XP_014094554.1\|odorant receptor 94a-like [Bactrocera oleae] |
| *BminOR40* | 273 | 90 | Unigene26536_All | 5'lost | 1 | 5E-41 | ref\|XP_011192526.1\|putative odorant receptor 85d [Bactrocera cucurbitae] |
| *BminOR41* | 200 | 66 | Unigene27188_All | 5'lost | 0 | 5E-36 | ref\|XP_011178699.1\|odorant receptor 33b-like [Bactrocera cucurbitae] |
| *BminOR42* | 201 | 66 | Unigene30659_All | 5'lost | 1 | 1E-35 | ref\|XP_011178699.1\|odorant receptor 33b-like [Bactrocera cucurbitae] |
| *BminOR43* | 269 | 89 | Unigene39195_All | 5'lost | 0 | 8E-50 | ref\|XP_011207940.1\|odorant receptor 33b-like [Bactrocera dorsalis] |
| *BminOR44* | 212 | 70 | Unigene39509_All | 5'lost | 2 | 2E-31 | ref\|XP_011191673.1\|odorant receptor 94a-like [Bactrocera cucurbitae] |
| *BminOR45* | 505 | 135 | Unigene42659_All | 5'lost | 2 | 5E-63 | ref\|XP_011184142.1\|odorant receptor 83a-like [Bactrocera cucurbitae] |
| *BminOR46* | 221 | 70 | Unigene45781_All | 5'lost | 0 | 9E-25 | ref\|XP_014101401.1\|odorant receptor 88a-like, partial [Bactrocera oleae] |
| *BminOR47* | 262 | 87 | Unigene46193_All | 5'lost | 1 | 1E-32 | ref\|XP_014094554.1\|odorant receptor 94a-like [Bactrocera oleae] |
| *BminOR48* | 200 | 66 | Unigene46619_All | 5'lost | 1 | 3E-14 | ref\|XP_014100962.1\|odorant receptor 43b-like, partial [Bactrocera oleae] |
| *BminOR49* | 249 | 83 | Unigene46769_All | 5'lost | 2 | 1E-35 | ref\|XP_011184142.1\|odorant receptor 83a-like [Bactrocera cucurbitae] |
| *BminOR50* | 218 | 72 | Unigene46882_All | 5'lost | 0 | 1E-37 | ref\|XP_011186833.1\|odorant receptor 59a-like [Bactrocera cucurbitae] |
| *BminOR51* | 1469 | 415 | CL8398.Contig3_All | 5'lost | 5 | 0 | XP_011210512.1 odorant receptor 7a-like [Bactrocera dorsalis] |
| *BminOR52* | 272 | 90 | CL8529.Contig3_All | 5'3'lost | 2 | 2E-53 | XP_004520354.1 odorant receptor 74a-like [Ceratitis capitata] |
| *BminOR53* | 2385 | 274 | CL2775.Contig4_All | 5'3'lost | 3 | 4E-169 | AKI29033.1 odorant receptor 13a [Bactrocera dorsalis] |
