# Supplementary material for: Identification of olfactory genes and functional analysis of BminCSP and BminOBP21 in Bactrocera minax
Source: PLoS One. 2019 Sep 11;14(9):e0222193. doi: 10.1371/journal.pone.0222193 (PMC6739056; doi:10.1371/journal.pone.0222193)
Supplement: S3 Table — (DOCX) [file pone.0222193.s003.docx]

**S3 Table** Unigenes of candidate ionotropic receptors

| Gene name | Length  (nt) | ORF (aa) | Unigene reference | Status | TMD  (No.) | Evalue | BLASTx best hit |
| --- | --- | --- | --- | --- | --- | --- | --- |
| *BminIR1* | 2620 | 674 | CL344.Contig2_All | Complete ORF | 3 | 0 | gb\|AKI28988.1\| ionotropic receptor 76b [Bactrocera dorsalis] |
| *BminIR2* | 750 | 250 | CL1784.Contig1_All | 5'lost | 1 | 3E-175 | ref\|XP_011207794.1\| lutamate receptor ionotropic, kainate 2 [Bactrocera dorsalis] |
| *BminIR3* | 4320 | 863 | CL1822.Contig2_All | Complete ORF | 3 | 0 | ref\|XP_011214731.1\| glutamate receptor ionotropic, kainate 2 [Bactrocera dorsalis] |
| *BminIR4* | 2799 | 864 | CL3025.Contig3_All | 5'lost | 3 | 0 | ref\|XP_014092137.1\| glutamate receptor ionotropic, kainate 2 [Bactrocera oleae] |
| *BminIR5* | 3210 | 966 | CL3650.Contig1_All | 5'lost | 3 | 0 | ref\|XP_014086336.1\| glutamate receptor ionotropic, kainate 2 [Bactrocera oleae] |
| *BminIR6* | 3735 | 989 | CL4168.Contig2_All | 5'lost | 4 | 0 | ref\|XP_014091080.1\| glutamate [NMDA] receptor subunit 1 [Bactrocera oleae] |
| *BminIR7* | 517 | 171 | CL6000.Contig1_All | 5'lost | 1 | 3E-63 | ref\|XP_016940247.1\| glutamate receptor ionotropic, kainate 5-like [Drosophila suzukii] |
| *BminIR8* | 354 | 104 | CL6828.Contig2_All | Complete ORF | 1 | 1E-43 | gb\|AMH85979.1\| ionotropic glutamate receptor, partial [Bactrocera cucurbitae] |
| *BminIR9* | 2163 | 639 | CL7699.Contig1_All | 5'lost | 3 | 0 | ref\|XP_014088063.1\| glutamate receptor ionotropic, kainate 2 [Bactrocera oleae] |
| *BminIR10* | 987 | 300 | CL7904.Contig1_All | 5'lost | 1 | 7E-174 | ref\|XP_014100381.1\| glutamate receptor ionotropic, kainate 3-like [Bactrocera oleae] |
| *BminIR11* | 1821 | 548 | CL8103.Contig1_All | Complete ORF | 1 | 1E-177 | gb\|AID61280.1\| ionotropic receptor, partial [Calliphora stygia] |
| *BminIR12* | 798 | 266 | CL8750.Contig2_All | 5'lost | 2 | 0 | gb\|AKI28987.1\| ionotropic receptor 75d [Bactrocera dorsalis] |
| *BminIR13* | 2990 | 956 | CL8940.Contig2_All | 5'lost | 3 | 0 | ref\|XP_011211753.1\| glutamate receptor ionotropic, kainate 2 [Bactrocera dorsalis] |
| *BminIR14* | 3551 | 442 | CL9351.Contig2_All | 5'lost | 0 | 0 | ref\|XP_011214752.1\| glutamate receptor ionotropic, delta-1 [Bactrocera dorsalis] |
| *BminIR15* | 234 | 78 | CL9877.Contig1_All | 5'lost | 0 | 3E-45 | ref\|XP_011207063.1\| glutamate receptor ionotropic, delta-1 [Bactrocera dorsalis] |
| *BminIR16* | 1188 | 395 | CL11683.Contig1_All | 5'lost | 3 | 0 | ref\|XP_011207794.1\| glutamate receptor ionotropic, kainate 2 [Bactrocera dorsalis] |
| *BminIR17* | 3999 | 990 | CL11993.Contig1_All | Complete ORF | 5 | 0 | gb\|AMH85977.1\| ionotropic glutamate receptor, partial [Bactrocera cucurbitae] |
| *BminIR18* | 1479 | 364 | Unigene7888_All | 5'lost | 0 | 8E-165 | gb\|AID61275.1\| ionotropic receptor [Calliphora stygia] |
| *BminIR19* | 2125 | 628 | Unigene10094_All | 5'lost | 3 | 0 | ref\|XP_011214726.1\| glutamate receptor ionotropic, kainate 2 [Bactrocera dorsalis] |
| *BminIR20* | 3163 | 936 | Unigene10095_All | Complete ORF | 3 | 0 | ref\|XP_011177766.1\| glutamate receptor ionotropic, kainate 2 isoform X1 [Bactrocera cucurbitae] |
| *BminIR21* | 608 | 202 | Unigene19848_All | 5'lost | 0 | 6E-138 | gb\|AKI28995.1\| ionotropic receptor GluIID [Bactrocera dorsalis] |
| *BminIR22* | 243 | 80 | Unigene26474_All | 5'lost | 0 | 9E-45 | ref\|XP_014101473.1\| glutamate receptor ionotropic, kainate 5-like, partial [Bactrocera oleae] |
| *BminIR23* | 233 | 77 | Unigene28486_All | 5'lost | 0 | 6E-39 | ref\|XP_011207794.1\| glutamate receptor ionotropic, kainate 2 [Bactrocera dorsalis] |
| *BminIR24* | 239 | 79 | Unigene28847_All | 5'lost | 1 | 1E-40 | ref\|XP_011176721.1\| glutamate receptor ionotropic, kainate 2 [Bactrocera cucurbitae] |
| *BminIR25* | 2309 | 654 | Unigene31517_All | 5'lost | 3 | 0 | ref\|XP_011195022.1\| glutamate receptor ionotropic, kainate 2-like [Bactrocera cucurbitae] |
| *BminIR26* | 2111 | 588 | Unigene31518_All | 5'lost | 2 | 0 | ref\|XP_011195022.1\| glutamate receptor ionotropic, kainate 2-like [Bactrocera cucurbitae] |
| *BminIR27* | 283 | 94 | Unigene37212_All | 5'lost | 1 | 1E-59 | ref\|XP_014089631.1\| glutamate receptor ionotropic, NMDA 2B [Bactrocera oleae] |
| *BminIR28* | 240 | 80 | Unigene46549_All | 5'lost | 2 | 8E-41 | ref\|XP_016979349.1\| glutamate receptor ionotropic, delta-2 [Drosophila rhopaloa] |
| *BminIR29* | 288 | 96 | Unigene46621_All | 5'lost | 0 | 9E-51 | gb\|AMH85979.1\| ionotropic glutamate receptor, partial [Bactrocera cucurbitae] |
