# Supplementary material for: Identification of olfactory genes and functional analysis of BminCSP and BminOBP21 in Bactrocera minax
Source: PLoS One. 2019 Sep 11;14(9):e0222193. doi: 10.1371/journal.pone.0222193 (PMC6739056; doi:10.1371/journal.pone.0222193)
Supplement: S4 Table — (DOCX) [file pone.0222193.s004.docx]

**S4 Table** Primers used in the qRT-PCR experiments for expression patterns of candidate olfactory genes

| Gene name | Direction | Nucleotide Sequence |
| --- | --- | --- |
| *Bmtubulin* | F(5'-3') | CAATGGCTGTGGTGTT |
| *Bmtubulin* | R(5'-3') | GTTGTGCCCAAGGATG |
| *BminIR14* | F(5'-3') | CTGTCCCTTGACCTTACC |
| *BminIR14* | R(5'-3') | ATACCGCCGTGACATCC |
| *BminCSP* | F(5'-3') | CAAGCAGCCTACGACACG |
| *BminCSP* | R(5'-3') | GGCGAAACCCTTCAACAT |
| *BminOBP8* | F(5'-3') | CTCGTTGGGCAAAGTGGT |
| *BminOBP8* | R(5'-3') | GCAATAGGAAGGCGGTGT |
| *BminOR4* | F(5'-3') | CAAACGCTTGGAGACAGG |
| *BminOR4* | R(5'-3') | AATGGGTTCAACGATGCT |
| *BminOBP16* | F(5'-3') | GCGTCAAACAAGTTCCAT |
| *BminOBP16* | R(5'-3') | CTCATCATCGCTGTCCTC |
| *BminOBP13* | F(5'-3') | TTGTATTTCCCAGCGTTTA |
| *BminOBP13* | R(5'-3') | AGTCATCCTGATTGCGTAT |
| *BminOBP4* | F(5'-3') | GCCACCGAGCTAAATGTCAC |
| *BminOBP4* | R(5'-3') | TTCATTTGCTCAGCACCCGC |
| *BminOBP12* | F(5'-3') | CCGCTAAACATGAAATCCTC |
| *BminOBP12* | R(5'-3') | ATCCGAAAAGCCGTATTCTC |
| *BminOBP21* | F(5'-3') | TATACCAACGCGTCAGAATC |
| *BminOBP21* | F(5'-3') | TCGGTGTTGGTAAT TTGGC |
