# Supplementary material for: Identification of olfactory genes and functional analysis of BminCSP and BminOBP21 in Bactrocera minax
Source: PLoS One. 2019 Sep 11;14(9):e0222193. doi: 10.1371/journal.pone.0222193 (PMC6739056; doi:10.1371/journal.pone.0222193)
Supplement: S5 Table — (DOCX) [file pone.0222193.s005.docx]

**S5 Table** Primers used in RNAi for PCR

| Gene name | Direction | Nucleotide Sequence |
| --- | --- | --- |
| *BminCSP* | F(5'-3') | GCTTCATAGCCGCAACAGT |
| *BminCSP* | R(5'-3') | CCATCGCCTTTGTCCTTCT |
| *BminOBP21* | F(5'-3') | AACGGCGAGGAGTTCACA |
| *BminOBP21* | F(5'-3') | TCCGCTTCCACATGCTAC |
