# Supplementary material for: Identification of olfactory genes and functional analysis of BminCSP and BminOBP21 in Bactrocera minax
Source: PLoS One. 2019 Sep 11;14(9):e0222193. doi: 10.1371/journal.pone.0222193 (PMC6739056; doi:10.1371/journal.pone.0222193)
Supplement: S1 Text — (DOCX) [file pone.0222193.s007.docx]

**S1 Text** FASTA format of the protein sequences of OBPs, CSPs, ORs, IRs, and SNMPs identified in this study.

>BminCSP

FSN GKAIDFKSMLRFIAATVLICTVYYVATTSAAPHPPTTAAPLAASQAAYDT KFDNIDLDEV LGQERLLRNY IKCLENTGPC TPDSKMLKGF ANFRDTPRRY FNRLRQMLGE AEAWLNEGDA LLDRQSSGGL GAFGADIRSA RQLPFGLFGG EGQRRWRGET YGSAGY

>BminOBP1

FHTSVVVLSP AEKSQSNTKQ KSKMKFFIVI LAVVALACAE DEWVPKNVAE IKVIRQECIK EFSLSEEHIQ KLKNLEYPDE EPVRKYLLCT AEKLGVFCEH EGFHANRIAK QFKMDLDEAE VLAIAEGCVD KNEEGSSPDV WAYRGHKCLI DSKIGESVKA YIKKSADEAK KQ

>BminOBP2

VKLSNEKIRM KYFMLIVILA VVALVQADDW SPKTVDDIKK IREECMKQVP SSDEEFQKRK ENDYPDVESV RKYALCNSKG WGLYKEGKGF YPDRVAEQFK DDMPEDEIKA IVNDCDEKTK EETDDERCYH LLKCVMSTKL GDHIKDLVKR LE

>BminOBP3 PBPRP

RENLYGN GTKMVLTGTR RGQAFHAFLI VALSSSLTLM HVQAQEPRRD DKWPPPAVLK MAKIFHDICV EKTGVTEEAI KEFSDGQIHE DEALKCYMNC LFHEIDVVDD NGDVHLETLF NTVPGTVRNQ LINMAKECEH PEGDTLCHKA WWFHQCWKKA DPVHYFLP

>BminOBP4

RTTNRENFTKRISPKMNFREASQVLLLITAAYCTQMVNSAATTKAANQDVESDTDILRNCLREVGSKDLVGELQKVARYSKWTSEEVPCFTRCLASKKRWFDADESKWNKQQIADDLGADMFNYCRYELDRYNEDSCEFAYTGLRCLKQAELYT LETYKNILSC ATELNVTMKE LQKYAAFPTK EVVPCLFQCL AEKMHFYTST YEWNFDNWIKAFGPMRQDRTASNVCKAGAEQMKTRHKCEWMYEEYNCLER LNYNTDGSYP LETTTLSAVI TSKTTKAGKD ITEAS

>BminOBP5

TWT APQAMKGTALVFIIPFSALFANINADYEEKTEDDFLSAGERCFQRERLAA SYQHRFDNFD YPDEQPVHRY VHCIWTELKL WNDRTGFNVEHIAALYRDKA NTEVLVPILS DCNRNAQNDP ILNWCYKAFK CVLNSRVGQW FKEDVGRKLQ ERHVGNHVA

>BminOBP6

SLSRQKMHSRKTLLGTLLWIGFLLNLIWAQKELRRDETYPPPELLKELQPVHDSCVAKTGVTEEAIKEFSDGDVHEDELLKCYMYCVFEETDVLHEDGEVHLEKIL DKLPESMHVI ALHMGKKCLY PKGDNKCERA FWLHRCWKEA DPKHYFLI

>BminOBP7

SF EIQKGKSKMK FFLVILAVVT QTYAEDEWRPKNMAELNAIRQECLKEFPLS EEQLQKIKNF EYTDEEPARK VLLCTVKKLG VFCEREGYNA DRVAKQFKMD LDEAEALAIV EGCLDKNLEG SSADVWAYRG HECVVASKIG DRVKAYFLKS KK

>BminOBP8

RKRNQFTANA QSENQNSFSK TPTTNNKTLT NMKFFTIVAV FAFVAVAAAQ EGIKLSEEQK QKVHTLGAEC LTETGASEAA VRAVGKGDLS QVDDKVKCFA KCLQGKLGYF ENGQVNEAVV QSSLGKVVGE EKIKAIQAKCNGLKGTDDCD TAFLLHKCYV AENASTLV

>BminOBP9

IKPNSKMKFF IVILAVITLA YAEDEWMPKT EAELKVIVQE CLKDVPLSKE QLQKISSFEY TDEEPLRKYF LCTTKKVGVF SEHEGYHVDR VAKQFKMDLD EAEVAAIAEG CADNNIEGSS ADVWAYRSHK CVMDSKI

>BminOBP10

NYSCCATCSR KLFLVRAEKY CIMKSFINIA LFVVSCAVVL CNANDPEMRK QIEECNKEHN VTPKDYHDFM EGKLTTIPEN LKCSSHCVMM KQGIMDDSGK FKAEVAKAKM NDDKFSATVD ECKDLSGSTP CDTAMKITEC LLSHK

>BminOBP11

VQRRKTIKPKSKMKFFIVILAVIALVYAKDEWVPKTEAELQVIVKDCLEDFHLNNEQLQKYTTYQQPDEEPIRKYMLCNAKRVGFFSEHEGYRVDRVAKQFKLDLDEAEVAAIAERCADKNVEGSSVDVWAYRGHKCVMASKIGERLRVYIENLKKEA KKH

>BminOBP12

TSI RNPQSPLNMK SSIICCILAT VLLSLCTFGT DAALGRKPKK LTPELESKFE VLTAWIAYRL NLKHAKEACV GEYGFSDELA TNLVKIRVAN PSDNEKCYVN CLYNKLVFYK DNAINKQAMK ESLYEIVGEQ RLMNIVNGCLNAGGTNACDK VYKFHACASP EFDKVRSDIF LPDE

>BminOBP13

DILREHFQ QELSLLLRNK MNYIVAVLLA VLVAMATAEE YKIRNQDDLL KARKECMEAK KVPTEHIEKF KKFEFPDDEV TRCYIECIFN KFQLFSPTEG FKTQNLIAQL GQNKENKDAV KADIEKCADK NEQKSDSCTW AYRGFKCFIS KNLPLVQESL KKN

>BminOBP14

N YTMNTKQFVF LLLIYQYAFLKGVHALEVPKHMVSSVKKLTNLCLKETGAT PDLFKEFKTT GKLPNNQNLK CFMHCILDKI GLIDADNIVH LDSLFEVMPP DFVPLIENLH TSCGTQSGAD GCETAFLTIE CYLNTNPIIS KMVFVTLGD

>BminOBP15

NYFIFEKCMAIIAADTGSNPPHYSSLRVMAEAAIEDCYEDAAESVKVQITDESFDE IVKGSRTNLS RNAKCLRYCI MRKNGLLNED NSIDRENILQ IFQIIHPQIE KDFLLDVIQK CSREMDKQAD NCECAFVASS CILRELQVDG VTDI

>BminOBP16

VQADDWSPKTTDEIKSIRENCVKQVPSSDEEFKNRKKDEYPDIESVRNYVLCTSKAWGLYEDGKGFKGEHVAQQFKGDLSEDEIKSIVHDCDEKTKEDSDDERAY HVLMCILSSK LGGDVKELLK RSE

>BminOBP17

L NMKFCLALLS LLMVVVFAVA DHAGHTDYVVKTNEDLIRYRDECVSKLSIP SDLVDKYKAW SFPDDEKTRC YLKCVLEKFE LFDAAKGFDVHNIHHQLVGA NADHSDATHG AIENCAKEAA GDDACVRAYNGFTCFLKNNAQLVQAGVEKS SK

>BminOBP18

KGYVLDPYIG PLNPKESGLV RNVFLIFKQL GKQEFDEPIL NNTNVAGHER FSSKGFAKKY DMELVAGNIF QSRWDEYVTL LHKQFGIIK

>BminOBP19

NFRSHPVQLG TILANSNIFT KYFPTYSTNM AKFILFAALC ILSAAVSNAA FNKEEAIKNF MTRAEECRGE VGAADSDIQD IVAKVPASSK EGKCLRSCLM KKYGAMDSNG KFVKSVADQH AQDFTDGDAD KLKTAREIIDACADIAVPDD HCEATEVYGK CFMDQAKAHG IQKFDF

>BminOBP20

VRVFAHWKVC LLLFLHGAYNLLRMYQFGVHEKRAATTTTSAVVKRSDGGK GMSLPGLTWL MFLLAMVVCV LPPGAVTLTP TAPSRSFVEACQVKHNVTME ELDEFPTDPS PEDIDMKFKC YADCLLNGMG FMDTNGKLNAEAMHEWGILN DESYKDILEC KAANDMEDDP CEYSFGMMLCARMLDSEEEYYSGEMDDEAK EERRK

>BminOBP21

AAQGEVEGVY YLRFWKMWQRILCVCLAASCAFAVDNEVEKLFRDMEVVPD ILDKPPKEKL KIEYNDGLDV GNGEEFTPTQ TKDEPKLYWT SEPDAYYTVI MVNPDIPTRQ NPLLREWLHW LVVNVPGDDV AKGDILDPYI GPMAPKMSGL LRYVFLIYKQ PGKQEFDEAK ITNTDVAGHE KFSSMGFAAK YNMELVAGNL FQARWDELVP KLHKQFGIVI

>BminOR1

TYTLTV LPCKSMAYWA IATRKGQSPP MKITPVLNPN QREFLEDELL YREKLEILAE NNTISTDLFV RKFEDIDDPV LLDKHDSFYH TTKSLLVLFQ IMGVMPIHRN PQKPGMPRTG YSWTSKQVFW AVCVFSMQTTIVVMVLRERV NTFLNDSDRR FDEAIYNVIF ISLLFTNFLL PVASWRHGPQ VAIFKNMWTN YQLKFLKVTG SPIVFPNLYP LTWSLCFFSW GVSIAINLSQ YYLQPDFKLW YTFAYYPIIA MLNGFCSLWY INCTAFGTAS RALSASLELT LMSDKPAKKL TEYRHLWVDL SHMMQQLGRA YSNMYGMYCL VVFFTTIIATYGSLSEIMDH GATYKEVGLF VIVFYCMSLL YIICNEAHYA TQSVGLDFQT KLLNVDLTAV DSATQKEVEM FLMAITKNPP IMNLDGYANI NRELITSNIS FMATYLVVLL QFKITEQRNY SLKQSRAELL A

>BminOR2

LAYAVSYFLQ CCGAFHVSLL FLSGDLLLIS MVHLVNMHFG YLIYKIESFQ PTGTDADMKV LGPLMVYHNE MLNYAERIDN TFGLATLLNY VGSCLVLCLI GLQIAMGSEA

>BminOR3

MTANILGERF QRALRHIGPA AMVADYRALW LRLSKLTRDT GNATCYTFTF INLYLFFVIT LSVYGLMSQL SEGFGIKDIG LAITAIWNVF LLFYICDKAH YASFNVRTNF QKKLLMVELN WMNSDAQTEI NMFIRATEMN PSNINCGGFF DVNRNLFKGL LTTMVTYLVV LLQFQISIPSDSGRHMNVSV AELATATMME SAEIELTTST TSTTTTTSTK MPPPARGRKG

>BminOR4

LQVS FYGAVLFFLLFHQIVNDSYPAIYIYIIRTQVQLLTNRVKRLETGNKSQEE KYHELLNCID THQEVLSLVS IVEPIMPLTM FVQFFIAAAI LRITMINIFI FANFATRIAS LTFLFCVLLQ TS

>BminOR5

KKLRATQIL TQRLDKRCRA SDEVEELRQM VRFGKKVVIF YLTIFLCYSA STFLASVSSG YPPYSLYFPF LKWRRSRTEF IIASLLEFII MDFACLQQTV NDGYPVIYIN MLRCHMKILQ FRVEKLGTNP TLTQVEHLSE LKLCIKDHQL LIELYDTIAPIISITLFIQFALSAVCIGTALINIVIFANEFQTQVACSFFILAVLIEIYP ACYFSQCLIN ESDKLADVIF HSNWIEQSPE YRKLIIFFLQ RSQRPMFLTA GKLFPVTLSS FVSIAKFSFS LYTFIEKMNL KERFGIE

>BminOR6 ORCO

TKVH QHNMQPSKYV GLVADLMPNI RLMKYSGLFM HNFTGGSGLF KKIYSSVHLV LVLVQFLLIL VNLALNAEEV NELSGNTITV LFFTHSITKF IYLAVSQKNF YRTLNIWNQV NSHPLFAESD ARYHGIALAK MRKLFTLVML TTVASAVAWT TITFFGESVK FAFEKETNST ITVEIPRLPI KSFYPWNAGA GMFYIISFAF QCYYLLFSMV HANLCDVLFC SWLIFACEQL QHLKGIMKPL MELSASLDTY RPNSAALFRS LSANSKSELI NNEEKEPTDL DISGVYSSKA DWGAQFRAPSTLQTFNGMNGTNPNGLTRKQEMMVRSAIKYWVERHKHVVR LVAAIGDTYG GALLLHMLTS TIMLTLLAYQ ATKITGVNVY AFTTIGYLGY ALAQVFHFCI FGNRLIEESS SVMEAAYSCH WYDGSEEAKT FVQIVCQQCQ KAMSISGAKF FTVSLDLFAS VLGAVVTYFM VLVQLK

>BminOR7

IVTWDEVFVV LISQLCMYYE YLGKLLEEMKVQDAMDAKKINAFYKQLHDY IYMHQYLNNL AVDLNDLFNF SILFSDAGIA ISICFNLVLI TDATDYLQMI TYASPLFVEV WLIYDAAKWG TLLETVTARI NEILYEQKWY DSSARFGKYT MMWIQSTNEP FRLTAFNMFY VNMKHFQDMM MLAYQLLTFL KS

>BminOR8

LLFTHFLLPV ASWRHGPEVA IFKNMWTNYQ YKFWRVTGTP IVFPNLYRLT WGLCIFSWTL SVAVNVSQYY LQPDFEFWYT F

>BminOR9

HTLYTM FDLIKGRGRT VFASRDAVIY LFNSFRYLGI NPPAKYRLPY FMYSAIITFF AVLFSPVIFN VGWLRDRNKL SVMEILTCVQ ASLNVMAVPL KCITLAMAQK RLRGIEPMVT ELDERFPTSE DKAKIKKCAV TGNRLVFGFA VSYFMYETLT VVSALVGGHA PLSLWIPNVD WHRSTWEYWL QVSFDAAVLF FLLYHQVLND SYPAVYIYII RTQVQLLTSR VEKLGYDEQK SVDENYQELL ECIVIHQKIL KIVKIVESVV SITVFTQFLV AAAILGVTMI NIFIFADLTT KIASVTYFFC VLLQTSPTCY HASYLLDDCD QLRIAIFQCN WIAQNKRFNN LLIYFLHRSQ DSMPFFALKL VPINLATNLS IAKFSFTLFT FIQEMGLGEN LKG

>BminOR10

PKPSKDPKNF RFPLQCIWLK LNGSWPLKPK VTGEFEKYFR LLYTTWAWYV VAMVGITIGF QSAFLLKSFG NIMVTTENGC TTFMGVLNFV RLLHLRLHQR DFQQLLAQFV KDIWITSSSH PIVERACARN MRVFQVISVL QSSLITMYC

>BminOR11

DEMSEIY SMKHCLKYPY FTLDLAATEP FTWSGARTYS YRRIWLRRAL FTFGAINLVY QNIGMLIYLF MPHESSAQST IVQVTETGGI MGLTMVGTSN MLVMFWYGDR IAMLLEKFQQ LFPTARLQRK AKFTKQSLRG VEFPHRIEHF VLKSNKLMKL ATTLYMFAFA YYNSLPIVEF LYEWTTPGIV WKYRYQSNTW YPWQNERNAK SFASFTLAYV CQVQSSLTGV AFIMAAEFML CFFTTQLQIH FDYLANALET IDAAGANANE DLKYLINYHS QLLSYSKETN AIFNVSFMVN LCTSAIAICL MGFSMVMISL AHAFKYSIGL TSFIVFTFFI CYTGKELTET SDKLLNAAFY GNWYDGNLAY RKMILFFIVR CRIPTELRAY KFTTVSMPTF TAILRSSYSL FTFFQAMGQ

>BminOR12

PVMVQLSLLQ ATVNVLGLPL KAIVITIFQT HLRSAEPIFV RLDERYQSTE SREQIKNCVA LSTRLFTIVG FMYHLYGGIT YFQALVTNNY PLRTWLPFTD YIPQPTIRYW AHFMFEVFHM AFLLSVQFTM DVFPAIYIRN LRTHLNLLTE RVSQLGGNPD FTDEQNYDEL VDCIVTHQELLEATNIIGSC CSITLFIQFV VVAIALCISM LNFFVFANRQ QQVSTLIYYL VLILQIMPTC YQASMIEEYS TKLPDAIFHC NWLAMDKRCR KLTIYFMHRA QENVTFVALK LFKINLTTNL SIVKFGFTLY TFMNNMGFGQ NLKELLE

>BminOR13

VWQVIKMVGH IYGASVVGIL LLTTILVIKT NEKIYVMHFF IPGVDVETSF GYLLTTAVHT VVFLAGAFGL FAGDLFFLIY LGQPELFRDI LILKIKELNE AAAQKDNKTE HLLINIIQWH QYYTDYNERC NEVFYYIIT

>BminOR14

NKHQIMSLYC QTLTHEIIRK IEILHQDFPI NRQLKKEIDD IMNAVWRNIR RIFLFYFFCC VGIIANYFFT ALFQNLYHHL KQTPNYEFIL PVPSQYPFWE KKGMAFPYYHLQMYMTGSALYVSGLGAVSFEGVFMVLCQHAVGLVKVHNL LVLRATSPQT PAERRLEYLR YTIFTYQRIN KYMHEIQTIF RHISLSQFLL SLIVLGFVLFEINYGLGSNIIIFIRLIMYISASITQITIYCYHGQALTTVNEKIPLAYYNCNWYGENKTFKQLIMMMIMRTNKEFYLEVSWFTLMNLATLISLIRASVSY YLLLQNFQEN

>BminOR15

ILLKAI ARKFEMRKIA DLFYGRGKDD FETTESFVLL FRGWAAVGFL PKIPKRIVDI IHQIICWCSI LTCPVWYFAG LIDMMDDLPI TLLLSNLGVA INCIALPLKA IYIKVNMNHL HDINLLFKRL DERYQTPEEN IQIRESVKIS TRIFAACCTL YWFFGISSGL VPLFAHEYPH GNVFPFIDWL PEGNFQYWLH YIVEMVNLQY LLHLQSINDS FPAVYIRNIR THIRLLTDRV SRLGLDPDLS DQQNFEELVD CIVSHQEILQ ISDTVGSILS LTTFFQFTIY AALICVCMLN MFIFGDLKVK LSTLIYLIPV IWQTVPTCYQ ASMLETDCSK LPEAIFHCNW LDLDKRCHKL IIYFMQRTQE EICFTAIKLF QINLGTNLSI AKFSFTLYTF IKEMGLDAHY NQK

>BminOR16

KYETKLFEWN QSMKNILIIG GLVYFVSAVL ALVTPIFLYI FKGERHLIIM CQMPYVDLDT DHGYFITIGY NILCVFVAAF GLYGADLYVF LFLTHSIFFY DIFALKVEDL HEVLHENKQD TRIKAMVNDI AGWHQYYLDF NDKCNQIFFW TITSHILCTI LGILTTLLII MLKYWPGAYP YIFVCFVWLY MYSILGTRVE ICNDQFCDGI YDINWYDLDV SDQKTVSLML MESQVPRIIT IAGIEPLSVN TALKITRSIY SLAMMVVQFN E

>BminOR17

TCVAFHKTYI EHLFFFTHSP LLSISIVSIN TVKEHIVRAH AVMAARLNFI PFVMGFITCT SYNLKPLLMT LILYVQGQKP MWKLPFNMTM PSFLLQAPYF PLTYIFTSYT GYITIFMYGG CDAFYFEFCS NIAALLELLQ NDLKSIISFG EDKITLTGEE STLLE

>BminOR18

PICFTVESFL ADNLNDFCEV IYIAMADMTL NIKFLTLFIV RRQLLELRPI LKRLDARAKT EEEMNVLQEG IDSAKKCFLI ILRLFYSAFV TSQLMVIFSA EARLMYPAWY PFDYQASRTK FWIAYGYQ

>BminOR19

RENFCFLCCLFVDIVFRDWDGIRLGLASSQGCVRIKKRAKMQKFSDVLYGRVE SDCDTNKPFK TLLHLYGLIG IKPKPKGFLP TLHMVIVWMA FGFTPLLSIV GFIRFQKTAT ITESLTRLQA VINAIFIVVK SLVVLVNLKR LQNVEPVMKS LDERYNTAQE RQQISDCVAA CTRLYASMGC LYYSYGTLSI LSALISHKQP FGVWYPFLDL ISNPTIYFYT CLLLEACYGY FLLAAQYLHD IYPTLYMRTL RTQIQLLRAR ISRLGEDPDM SDEENHKELV ECIDTHQKIL QVVDMVGSVC SPTIFIQFSV VAIVHCICMV NLFIFADTIN KVITILYYAT VGMQILPTCY EASTLEMESS KLPDSIFHCN WLALDKRGRR LITFFIQRAQ VEVSFVAIQM FEINLRTYVA IAKFSFTLYT FVNEMGFGQN LKQLME

>BminOR20

QVVPRAAG VFWKHIKPIS IFIYYLITFL YFQCDGNEIT LKKVHEFMRI VKKASKTILI LTALTLFYIT VIQLLATADY EHKRLFVDVA IPGINLYKSP FYEMMTALQI LVLVPLTLPS YVSYLCLKLT CISFGTFLIK DLQYKLESTT DMTELEALKC IKKCVKDHVM IIKYHNDLEV LFSSGSSVSV SIFGITPCVI IVFSTMVSSN RSNLFLCYIF KFKFTIPTLY HRNFFIYFTY LALTGPRYVI VDCRHSTVSS SHDFDIYILL GGQ

>BminOR21

QKLLEWNKSM KKILFACAIV YSITAMLILS TPIVMYILKG ERHLILLCEV PGFAADSYYG YWVNNAFNLL CVMIAAFGLY AGDLYLLLFL THSIFFYDIL VLKINDLHKL LEHEDKEDRQ TRIVKDIVEW HQFYLDFNDT CNLLFFWTIS AHIICTTTGI LSTLLIIMLK DWPGAYTYLF VCFLWLYMYC ILGTRVEISN DQFCTGIYDI HWYALDVHNQ KTIRLMLTQS QAPRNITIAG IEPLSVNTAL KITRSIYSLA MMVLRFQTK

>BminOR22

ELVTLYDNIQ DSGDDYALSV LAAATKSARN ISIFNLSASF SDLVVAMAYP LFRQQRVHPF GVALPGIDVT HSPLYEIIYI GQLSFPFTLS SMYMPYVSLF A

>BminOR23

KMDYFVPL QFDNRPIKLP IQVAGYKFNF LWPLKEDAGI LSRLVNNICV SVSVLCYIGT IVGEFTFIGE NIADIAAVAE CLCTSFMGVQ YIIRIFVLLS RQRALRKLLR NFYRDIYFTP ADDAGLYKEI NSIMRFMNIF TQFYYVPMML ILVLYVYDV

>BminOR24

SEGIDSIIRN SYMLVLWFNT ILRAYLLLYD REKYEKLLSD LETFYYDLKR SKDSYIQDLL VEVNTTGKYM ARGNLFLGLL TCFGFGFYPL FATERVLPFG SMIPGVEEYK SPFYEFWYIY QMVITPMGCC MYIPYTSLIV AFIMFGIVMC KALQFRLKTL HRVRHIESLI HKNVRECIRY QLSIIDYIAR VNALTTYIFL LEFLAFGT

>BminOR25

NIKIFLKLGL IGSGARTMRV LLGLVLILTY SGQLINFCKT WNEDIGESGM NFHVVALIMH SVVRFFVVLK KDKKFERFFQ RTEQWFTDIE RNGDPQIVST LQNITKKTQK QTKMIFCAGA VGTLAGFIYS VSFDKRKLLV A

>BminOR26

TYNCSTKHFASNMQYYWTATQGDCHYFARLDARYQSVASREQIKNCVVVSTRL LASVGFMFNL YGSTTYLQAL LTRGYPMRAW LPFIDYIPQPTIRYWVHFIF EVSHMAFLLT LQASMDAFPA IYIRNLRTHL

>BminOR27

TSLERKTMPEDLFRIQRNCLRVMGHQDIFDNNEASSSDEQKSKSKRQRRCFRHWQALKYVLLLLFMVSAQLPMMNYIIYHIDDLALATACLSIVFTNVLTVIKTST FLTYKREFKS LMAEFESMYD E

>BminOR28

RAMNAIERNT NFTRFTAGPV RYFKFLGILL QQPEMPHSKY QRLLIVVTIA LMFLHQIGYI LEPGRTFAEQ SAAAGLLNYT TVSGGKILFL VYNRRLLLSN HCQLAALYPS

>BminOR29

LLFACFCSFC AGLIILSADL CLFSSVSQLM LHLDLLAQRI KELQPAEEGS LSALKAIIEY HQKILTIAKD VNSIFAPSIL FSLASSSFIL CFSAYQLLDD VSFIFALKVF

>BminOR30

KDEMKRNYDE LVSSIKDHKT IIELFSTIQK PISGTSMAQF VCTGVAQCTI GVYMLYVGFN ISIMLNMAVF FVSVTMETLI LCYYG

>BminOR31

ELHEQNVYSW QHIQEIVEVT NAMAPFMQAT ISLWKIWRVIYRRKEMAQMA ENIYLISTRA SAKELTHLIQ ENNRERLMNT AYYYSVLNTG MLALAAPVLV SFIQYLRLGE FSYIVVLKAT YPIEYARPLN YLLIWLWTAV AIYGVIYGSV SVDSLYSWYI HNLVGNFKIL QSKLVTAESA SELSERRELI YYCIAYHQRI IAMTEQLNII YQPIVFVQFS LNALQICFLA YQIGSGVVD

>BminOR32

LDVADISLSK IYCNMQTKTR PSDNFCKLLK IIRLSSSLVG IDVIDENFKF NYVVGFVLVA IAWNFTISIY TIWKDVKTDW TVLLDVFSPI SCATQGVVKI ISILLYPKLY RELAMDLVNI YKK

>BminOR33

HYDHISRSLEGYQTKFAEIHGKNGLTQQARAAMELKAVKDDIKFISSIVAYHTELLSLSDLLNKLYGLPLFVNFFTSSAIICFVSFQMSITREFDLLVKL GLFLFFSLLQ VYLICYFGQL LID

>BminOR34

HNENETTMLN EFKECIAYHA KIIDLCDDLE DLISIDGFFH LALFGLMLCM LLFFLSMMHY LQHIITALGF IVFDIYLLGV SYYYANKLAIESVEVTNAVY GTPWYRGNME MRKCVNIMMS RCQKPLQITA GGLYLMTMENFQAILRLSYS YFSLLRGFNQ Q

>BminOR35

RVEKLGYDDQ KSADENYQEL SECIVIHQKI LKIVKIVESV VSITVFTQFL VAAAILGVTM INIFIFADLT TKIASVTYFF CVLLQTSPTC YHASYLLADC DELRIAIFQC NWIAQ

>BminOR36

AEERAAYQLV PYFRTINISN KYLSIWHLSI TSIFVVHPLI ASIHGYISRE DKNESFDFTL PFMMTYFYDI NQPLAYAVSY FLQC

>BminOR37

KRFVKLYFGA CTSTGLYFTI NPLVSMIWAK FQAKPIPLEL PMPMRFPFDF ESTPGYEFAY IYTVFITIVV VMHATSVDGL FVSFTTNLRG HFQALQYFIE TNTFDKSEAL LQRELGIYVQ YHVRLLGLAQ SVQRIFKPII FGQFLMTSLQ VCVIIYQLVM NMGVIMEMVV YCTFLSSILL QLLIYCYGAE FLKTESSAVS TAIQMSQWYN LPPRHRHVLR LMMLRSQREI IISAGFYEAS LANFMSILKA AMSYITFIQS IE

>BminOR38

RLGTNSDFND EQNFRELVAC ITSHQLILQV ADIVGSILSL TVFLQLAFAA AILCVCMLNI FIFADTMHKV TTIVCYLVVL MQTVPSCYQA SMLEADCAKL PDAIFHCNWM DMDKRFRKLI IYFLHHTQTE ITFTGVKLFR INLSTNLSIA KFSFTLYTFM NKMGFGKNVK ECLE

>BminOR39

TLQTSMA GKKCSTNSPP SMSRIAAARF LVATLKAIGL WEWRMDSSRL GNTRLPCRLK LQRIYGLILH IPFTFIYITL MTMAVLCSQN LEEISTVLHF LLTEFSLVVK ISHIWQYSGA AWRYMAELAN EPIYALRHQS

>BminOR40

KRRSVQTRLA TVLRQIFSFV SLANLTWVLI IETLFVVVNF VENADFLEAA RNFTFMGFVI VAIIKYLSNL KQRSRLSVLM QKLYEIYPKQ

>BminOR41

ANMAAISATL LSKERRLLYP AWLPFRWEAS TFNYCAAVIY QIAGVTIQIV QNLANDIYPP MSLCII

>BminOR42

YIYYAVYFVS MAIELLPSCF YGSMLIYEFQ QLPSAIFKCS WLGQSREFYQ NQRIFVQVSL KKIVPL

>BminOR43

VGVSLQITQN LAHDTFAPVS LCVMSGQVRL LAMRVSKVGY DESKTLAEHE EELNECIEDH KKLLRIFDLL QDVFWYTQLV QFSSVGLNI

>BminOR44

GSCEQIISLSMLTQIMLSFLIICFIIYNLQSVHFSENPVHCLAMLQYVLIVSLQMFLPCY YANELTVESE

>BminOR45

SLYNKIWLLK TIEVTFAMCM CAFHLVKSSN DTQFLQVLCL GQYTVLGLLE IFMICYAGEI IYVNSQRCDE ALLRSSWYPH LREVRADFLL FLIHAQRSFE LTAGKFNPLR LDKFRGIITT SFSFFTLLQN LDERN

>BminOR46

FIATTTM ALQQERNGAA KVCAIDDLCA ILHPAQRYLG LNYLNFRRVN GRFAIPRSKL LNIALFLAVV DCT

>BminOR47

ALRRPKRIGG VDADEPEIID QMSDIFRMHM NVKRLTTQCE KLVSIPVLSQ IVLSAFILCF SGYRLQHNIA DIGIVISTIQ FTSVMIL

>BminOR48

TRNMRKLTDL LYGRGAAKFE SNESFQLIFQ CWSLFGIKPL KQYRSGRLLH MCFCWFCLIL CPFSFY

>BminOR49

KMFASSCFLA VIFRIILPII YNERTLPLTC WYPVDYKAPI IFEIAYLFQI LAQLQLSAAF SVSSAYFVAL CFLLSGQFDV LNC

>BminOR50

AITSELTLLI MGFAYEWRLM YPAYFPFDPY ATKAGYVTAH VFQIIGLMVQ LAENLVSDTY GGMCLTLLAG HA

>BminOR51

RPNRFAAIAS INIFLFKLAL VMSKILRVSR AKIYKSRDAV SYLFNIFTFL GTNPLEHRSR RYYFLYYFYS FTLNFISLLY CPLSFHIGYI KLTHVLTNSQ LLTAIQNAIQ VSGIPIKVVA ITWYMKRLQH AVEILDELDV NYTQREDLAK IRECVRRCRK VILLFCLPYY SFGISTIALG VLQNQAPLTV WVPFLDGKRA AWEYWTIVLW DTLVMFILLC HQLGNDTYPP IFIKVISTHM QLLVTRVNRL GRPGALTADK HYEELLACIR SHVHIVSIAK IVAPVISVTL FTQFATTATT LLNWFGDMEY PENIISLAFF CCQLVQILPC CLSASQLIAD CERLPDAIFH CNWMDQDRRF RRAILFFLQR TQTPIRFSCL KLFDANLETS VAIGKFAFSL YTLIHEAEDG GKTDN

>BminOR52

KIRRQHLPTK YSTFTYIITL VTYVYVPISG LVKNERLLPF PIKFGFDYTV PWPRYVVFLA MSIWTGFAVV GPLIGEANML AMQILHLNGR

>BminOR53

LYMLTVNTEP DILDSVPKPF PYKMLFPYDA NHGWRYALTY LFTAYAGICV VTTLFAEDSL FGFFAAYTCG QFRILHTQID NIIPDSYAAT RAGRGTEAVY QRECVRRLDR IAEKHCVLFN FVSRMENFFS PILLVNFLLS SVLICMVGFQ LVTGKNMFIG DYVKFLVYIL SSLSQLFVLC WNGDNIIQNS LEMANHLYAC DWECGVKLAAANANINNMHPDEQMKNLAPIVYYSTNTVFRKKLQFMIMRSHRQTCITAMK FSILSLNSFS GVCT

>BminIR1

KKKINTFVESSAH FAMTGFDLIL SAALCLTCAN LTDIRLPEGLIELDENNTVV TISPDLAVDE PSLDDAPLET VKTIIAKKEK MDKLREWIKG RKLVIATLED YPLSYTVMEN DTRVGKGVAF ELIDFLQEQM QFTYEVVVPE DNIIGSREDY EKSLIKMLNN SEADLAAAFI PTLSEQHSFV FYSTTTLDEG EWIMVMQRPR ESATGSGLMA PFDFWVWILI FISLLAVGPI IYMLIILRNR LTGDKEQKPY SLGHCAWFVY GALMKQGSTL SPIADSTRLL FATWWIFITI LTSFYTANLT AFLTLSKFTL PYNTVSDILY KNKHFVSARG GGVEYAIRNT NESLSMLTNM IRNNHAVFSS SSNDTFNLQN FVEKDGYVFV RDRPAINHVL YADYRYRKTI SMNDEKLHCP FAMAKEPFLK KNRSFAYPLG SNLSELFDPK LLNLVESGII KYLSTKDLPN AEICPQNLAG TERQLRNTDL MMTYYIMFAG FVTAMVVFFT ELIFRYLNQR NEGSKWARHG VGRTTNGLSV RAPRWLRQLE TDSDKQRLTA SPSGSTITPP PPYQSIFSSN HRHHQQDEAG HLSKESSLHR WRRAGQFGAG GSNFGTLAAG AGSGAGVLLG NGQLHDGSGAGGVRRLINGRDYMVFRNPNG QSQLVPVRAP SAALFQYTYT E

>BminIR2

IDQDKVKYGT LAGGSTSVFF SESNETEYKR AWNQMISFKP SAFTSSNKEG VDRVRKGNGS YAFLMETTTL SYNIERDCQL KQVGSQFSEK HYALAVPLGA EYRSNLSVSL LQLSEKGELY NLKHRWWTPT VKPECPSEDE ADGDELSIIE LSGVFLVLGA GVVVSFIIGC CEFLWNVQTV AVNEKTTPWQ AFKAELCFVL KFWITKKPAI ISESTKSSIS SNSSSKRSFD RSHSRSHSRV SGRSRRTKSK

>BminIR3

SVKMQSA VSPRWCWRYL RLSCCLAVLG ALLQHTLALP DVIKIGGLFH PTDDTQELAF RQAVEHINSD RLILPRSKLV AQIERISPFD SFHAGKRVCG LLNIGVAAIF GPQSSNTASH VQSICDNMEI PHLENRWDYR LRRESCLVNL YPHPNTLAKA YVDIVKQWEW KTFTIIYENN DGIVRLQELI KAHDNSPYPI TVRQLSLSGD YRPLLKQIKN SAEAHIVLDC SSDKIYEVLK QAQQIGMMSD YHSYLITSLD LHTINLDEFR YGGTNITGFR LINEKVVSDV VRQWSFDDKG LQRSANLSTV RAETALMYDA VHLFAKALHD LDTSQQIDIH PISCDGQNTW QHGFSLINYM KIVEMKGLTN VIKFDHQGFR TDFVLDIIEL GPMGIRKIGT WNSTLPDGIN FTRTYSQKQR EIEANLKNKT LTVTTLLSNP YCMRKESAVP LTGNDQFEGY VVDLIHEISK ALGFHYKIQL VPDGSYGSFN KQTLEWNGMI RELLEQRADL AVADLTITFE REQAVDFTMP FMNLGVSVLY RKPVKQPPNL FSFLSPLSLD VWIYMATAYL GVSVLLFILA RFTPYEWPAY SDAHGEKIES QFTLMNCMWF AIGSLMQQGCDFLPKALSTR MVAGIWWFFT LIMISSYTAN LAAFLTVERM DSPIESAEDL AKQTRIKYGA LKGGSTAAFF RESKISTYQR MWSFMESARP SVFTSTNAEG VERVAKGKGS YAFLMESTSI EYVTERNCEL TQVGGMLDTK GYGIAAPPNS PYRTAINGVI LKLQEEGKLH ILKTKWWKEK RGGGSCRVET SKSSSAANEL GLANVGGVFV VLMGGMGVAC VIAVCEFVWK SRKVAVEERL SAILHE

>BminIR4

AIDKANEESG GALELHGIAV AIEPGNAFET SKKLCKMLRQ NLVAVFGPTT DLAAKHAMSI CDAKELPFID TRWDFAVQMP TVNLYPHASQLAVALKDLVV ALEWTDTFTI IYETGEFLPT VNQLLEMYGT MGPTITVRRY ELDLNGDYRN VLRRIKNSGD YSFVVVGSMA TLPEFFKQAQ QVGLMTDDYR YIVGNLDFQT MDLEPFQHGDTNITGIRLVSPDEKLVQDLAKTLYETEEPFQNVSCPLTTSMALVYDGVQL LAETFKHVMF RAVPLNCNDA SSWDKGYTLV NYMKSLSLTG LTGEVKFDYE GLRTDFVLDV IELTMSGMQK IGEWKTEGGF FANRPPPKIV EVDQRSLVNK SFVVITAISE PYGMLKETPA KLEGNDQFEG FGIELIEELG KKLGFTYTFR LQVDNKYGSF NPKTGKYDGM MLEIIEGRAD MGITDLTMTS IREEGVDFTI PFMNLGIAIL FRKPMKEPPK LFSFMSPFSG TVWMWLGIAY MSVSLTLFIL GRISPTEWDN PYPCIEEPTE LENQFSFPNC LWFSIGALLQ QGSELAPKAY STRTVASIWW FFTLILVSSY TANLAAFLTI ESLSSPIENA EDLANNKGGV KYGAKVGGSTFTFFQDAKYPTYQKMYEFMRDHPEYMTSTN AEGVDRVENE NYAFLMESTT IEYITERRCS LTQVGSLLDE KGYGIAMRKN WPYRDMLSQAVLELQEQGVLTKMKTKWWKEKRGGGACSQEGENDGAEELG IANLGGVFFV LCVGSVFASV WGLLEWLRHVYGTARRNKVS FKTELIEEFR FVMQCSGNTR PVKYPKNSSR SRSRSSRSRS HSRSSSKSST LSVDSLPMDE SKLHHISEHT KHAK

>BminIR5

A LLPDKPTKTS MFFFINDADN EPAAKAVTVV STYLKKNPSY GISIQIDQVE ANKTDAKTLL ESICSKYAES IDRKQPPHVV FDTTKSGISS ETVKSFTQAL GLPTISASYG QEGDLRQWRD MDESKQKYLL QVMPPADLIP EVVRSIVRKM NITNAAILYD DTFVMDHKYK SLLQNIQTRH VITGIAKEGK REREEQIEKL RNLDINNFFI LGNLMSIRMV LESVKPTYFE RNFAWHAITQ SEGEVSSQRD NATIMFLKPM SYAQNRDRFG RLKTTFNLNE EPQIMSAFYF DLALRTFLAI KDMLQSGAWP KNMEYIGCDE FQGGNTPERN IDLRTAFTMI QEPTSYGVFE LVTQPGKSFN GYSYMKFEMD INVLQIRGGN SVNTKSIGTW TAGLDSPLVV KDEDVMKNLT ADTVYRIFTV VQAPFIIKDE KAPKGYKGYC IDLINEIADI VHFDYTIQEV EDGKFGNMDE KGEWNGIVKK LMDKQADIGL GSMHVMAERE IVIDFTVPYY DLVGITIMMQ RPQVPSSLFK FLTVLETNVW LCILAAYFFT SFLMWIFDRW SPYSYQNNRE KYKDDDEKRE FNLKECLWFC MTSLTPQGGG EAPKNLSGRL VAATWWLFGF IIIASYTANL AAFLTVSRLD TPVESLDDLA KQYKILYAPL NGSSAMTYFQ RMANIEQRFY EIWKDLSLND SLTPLERSKL AVWDYPVSDK YTKMWQAMQE AQLPATLEEAVERVRNSTSA TGFAFLGDAT DIRYLVMTNC DLQIVGEEFS RKPYAIAVQQ GSHLKDQFNN AILTLLNKRQ LEKFKEKWWK NDETQAKCDK PEDQSDGISI HNIGGVFIVI FVGIGMACIT LVFEYWWYKY RKNPRIVDVI EANSGGKDGK TIDSVILGQA GKEYDKGGNT VLRPRFHQYP TTFKPRF

>BminIR6

LM SNLAVFYGMSVVYLFFCGIH LGAIAQKHIQHSDNPSTYNIGGVLADAESE SHFRTIISHL NFDQQYVPRK VTYYDKTIRM DKNPIKTVFN VCDKLIEKRV YAVVVSHEQT SGDLSPAAVS YTSGFYQIPV IGISSRDAAF SDKNIHVSFL RTVPPYYHQA DVWLEIMFHF GYTKVIIIHS SDTDGRAILG RFQTTSQTNY DDIDVRATVE MIVEFEPKLD SFTEHLIDMK TAQSRVYLLY ASTDDAHVIF RDAAINNMTE GGHAWIVTEQ ALHANNTPVG VLGLVLEHAN SDKEHIRDSV YVLASAIKEM MSNETITEAP KDCGDSGVNW ESGKRLFQYL KTRNITGDTG QVAFDDNGDR IYAGYDVINI HEKQKKHVVG KFYYDPEKAK MRLRINDSDI LWPGKQKKKP EGIMIPTHLK ILTIEEKPFV YLRRLTDDEV SCDEDEIPCP LFNATDGSEN ENCCRGYCID LLNALSHRIN FTFDLALSPD GQFGHYTLKN VSSTSSGTIT SRKEWSGLIG ELVNERADMA MPLTINPERA EFIEFSKPFK YQGITILEKK PSRSSTLVSF LQPFSNTLWI LVVVSVHVVA LVLYLLDRFS PFGRFKLSHT DSNEEKPLNL SSAIWFAWGV LLNSGIGEGT PRSFSARVLG MFWAGFAMII VASYTANLAA FLVLERPKTK LSGINDARLR NTMENLTCAT VKGSSVDMYF RRQVELSNMYRTMEANNYDTAEQAIHDVKKGKLMAFIWDS SRLEYEASKD CELVTAGELF GRSGYGIGLQ KGSPWTDAVT LAILEFHESG FMEALDKHWI FHGNAQQCEL FEKTPNTLGL QNMAGVFILVAAGVAGGVGL IIVEVIYKKH QVKKQKRLDI ARHAADKWRG TIEKRKTLRA SLAMQRQYNV GLNAIPGTIS FAVDKRRYPR MGPRAPEQAW KSDADVLRNR RYLDDATKGG HSPAVHIPIL GKMRPPTNML PPRYSPAYTS NVSHLVV

>BminIR7

SLLATFLISF GSACSQGSDM VPGSMGGRMV FYTLYLLTFL MYNYYTSIVV SSLLGSPVKS DIKTMGQLAD SSLEVGLEPL PFTLTYLNNS LLPEVRRFKH KIDSVPNPKA IWMPLEKGIL RVRDQPGFVF GFEASTGFLL VKRYYKPYEI CDLNEILFRP EKSLYSAVHK N

>BminIR8

SWNIKKNMQPQNNWFLGIIYIFTLYVKGFANINRIEGISIGIISDHNTEQLRKTFDY AITVANSDLG IPLIGYNEEI KFGDSIEGYA KLCKFMQAGI GAIFGPS

>BminIR9

TQPIVQDLIS QLDYKENNDN RIVNGSCPIT LEMALTYDAV QVFAAITKKL IYRPQALNCS EESNQVQTDG STFKNYMRSI NIQERTITGP IYFDGNIRKG YMLDIVELQT SGLVKIGTWE ERSNLTIQRP PQLELWSEVD ANSLVNKTFR VLISVPNKPY ANLVESHKKL VGNNQYEGYS IDLIKELAAK LGFNYTFIDG GSNYGSFNKT TNKTTGMMKE INEGRADLAI TDLTITSERE EIIDFSIPFM NLGIAILYTQ PQKSPAKTFS FMDPFSVDVW IYLGFVYIGV SFCFFILGRL SPTEWDNPYP CIEEPEELEN QFTLNNSFWF TTGAFLQQGS EIAPKSLSTR TLASIWWFFT LIILSSYTAN LAAFLTIEKP IGLINNVNDL ADNTGGVEYG AKITGSTRSF FLTSEHEVYK KMNDFMAKNT HLLFETNLEG VNRVKSDSNY AFLMESTSIE YHIVRECKLM KVGEPLDEKG YGIAMIKNWP YRDKFNNALL ELQEQGVLAR LKNKWWNEVGAGVCKKKSDNSQVEPLDLNNLGGVYLVLGI GSGLSLIYGL IMWCIHVTRK SNYFEVPFGA AFLEELRIAI DVANNERILK SAQSVYSRSR NSLVSIDSID TDSEIEDSSK ADGESQKTI

>BminIR10

L NACKKMLNYW LIVAIICLSA KNAMALPHIN IGAIFYEDEL DLERTFIATV ESINSEKLNN FKMLPLIRRV SETDGSMILQ REACDLVDNG VLAIFGPSAK TDSDIVSLIC NATGIPHIQF DMCSEETDME SVNHQMTLNV FPAQQMLSKA YADIVQTYGW TKFTIVYDGD DPKAPTRLQD LLQLRDIHSD VVRLRVFKRN DDYRPLWKSI KGERRIVLDC GPEFLIDLLN TSIEFHLMGQ FNNLLLTNLE THSTNLEELR DNVTFAVNIT ATRLKLNGNS YYANAWQRAS MYDADPLSE

>BminIR11

DILSFARSFY NASRISVWRS LRNKFLFAYR KDLQQDTTAY FDDSLFIDQP NVLIVEAECG NCSTFALKTN KFIGPLAEHP EQLYVLDRYN GVDGKFELGV DLYMDKVQNL QGREVTVGVF DYRPFTVIDY ERQPQIKDHS PENPRGMAHI DGTEVRMLLA LCEVVNCTVN TDTSEDDWGT SYANLTADGI FGLVTSRKSH YAVGALYFWP DDYRYLDMSL FIGRSGVTCL VPSPHRLTSW LLPLRPFQPT LWLGVFACLG VEALALFFTR HLAPSDTEPQ YGLMESFQFG YITTLKLFVS QGSDYVVNSH TVRMVLFACY MMDTIVTSVY GGGLSAILTL PTLEEASDSV ERLYRHGIPW TATSPDWVIS LKGADDDPMV EKLLQKYHVY TYEQLTEFAK TENMGFILER LAFGHFGNVD FLTDESFKRL KLMIDDIYFQ YCFAFVPRLW ALLPKLNDVI MRVHSTGLDI FWEWEVAATYMDGQQQEEIQASMYMDFDVG PVKLDMGNFI GLVLPLIIGF VFSIFAFIGE LIYYKYTQKK AQAVINVN

>BminIR12

FAMPFASDVW IAILLLMIFT IGIFMVELIY SPHAHDIDIL DCVVFVWGAM CQQGFYANLL NRSARAIIFT TFVSTLFLYT SFSANIVALLQSPSEAIQTL SDLTQSPLEI GVQDTVYNKI YFNESTDPVT NHLYHKKIAP KGESIFMRPP VGMEKMRTGL FAYQVELQAG YQIISNTFSE PEKCGLKELE PFQLPMIAVP TRKNFPYKEL FRRQLRWQRE VGLMNREELK WFPQKPKCEG GMGGFVSIGI TECRYALGIF GFGLLL

>BminIR13

THREWTFRNC GIMWLLQRLV IFASFGYVCA NELKIAFWID PVQAGIELDV ASAIKEIEAL QLETKIQYYV VVIKNVGKKK EQKNMEKLCE HLATDGVSVV IDFTYHIWHN GLDLLRTYQI PFLRVDRILA PYFKMFSQYV MQKSGHECIM IFQNARDTEE AIIQIVEGYP YRSLVMNAYG KQDFIKRLRK IRPMPSCYAI FADGTAMNSI FDRISKENIF ERPREWHFVY LDPRDRVFKF KKIVDNATKF TINPKTLCRT LRMKDTYCLS GFSFQRAIIL EILRGLIELK QSNLYWLQSF VMECNYTSPN ENGTAGFDIL EQFPLNDFLY FTTDVTFPDD EFDHVPRLTY SPTININLYT SEHDAVTELA IWQNDNLRKI NETISPPRRF FRIGTVEAIP WNYMKRNPRT GELVLDAYGN PIWQGFCIDS IQKLSERLNF GYMLVPPTSG EFGRRDVVND IWDGIVGDLV TGETDFAVTA LKMYAEREEV IDYIAPYFEQ TGISIVMRKP VRQTSLFKFM TVLRVEVWFS IIAALVGSAI MIWLLDKYSP YSYRNNRAAY QYPCREFTLR ESFWFALTSF TPQGGGEAPK AISGRIMVAA YWLFVVLMLA TFTANLAAFL TVERMQAPVQ SLEQLARQSR INYTVVEGSS THQYFINMKF AEDTLYRMWK ELTLNVTEDF QKYRIWDYPI KEQYGTILLA INGSEPVKNA KEGYRKVNEH ENADFAFIHD SSEIKYELTR NCNLTEVGEV FAEQPYAIAI QQGSHFADEL SYALLELQKD RFFEDLKAKY WNMSRIKACS VNEEQEGISL ESLGGVFIAT LFGLGLAMVT LVLEIIYYRR KYSAMNRFTE ITKVKPTSGT SIKQLLPKKK SKKRIGIWHT STPKRDNSPE HKTPPPAFDA VKFRGKKVPP SITLGGQEFK PRRAGLRQLS ESLDSAEYRK EGIPARRDDD LPPYTE

>BminIR14

AMRLHASL WLLWLPLCLA LLPQNAEAND FSSFLTANAS LAVVVDQEYM QRRGENILAN FQKILSDVIR ENIKNGGIEV KYYSWSQIRL KKDFLAAMTV TDCKSTWQFF DSTQQNSILL IAITDANCPR LPSNRAIMIP IVDDGQELSQ IILDIKVQRL LRWKTAAVLL DQTILHDNPTLVESVVHESA KNHITPFSLL LYQIDDTLRS QRKRIAIRQM LNAFQGRTQT SRQFIVLSKF YEDIVEIAAS MKLFDVFNQW VFFVMNEEQH NHDPMSVTQN LEEGANIAFA LNTTEPTCSS SINCTITELSLAFVRSISRMIVEEQSIYGEISDEEWEAIRYTKQEKQDEILAYMKEYLRE YSKCTSCSHW KIETALTWGK SDEHRRYQSN LELRDTRNKN FEFIDVGYWT PTLGFNTHEV MFPHITHFFR NITLDILTVH VSCG

>BminIR15

SYVTSTAWVH ENKQFFVEAF DETAQEIGRK MEIVDNIDSV TERIAKGEYA YFDNEYFLRY LRMKGAQRRE EQQFTEVV

>BminIRl6

LKEDYENLRG MERYEGYAVD LIQKLANIMN FEYEFLIESR TGKKNPETGE WDGMIRRLID HQAQIAISDI TITQARRQVV DFTVPFMQLG ISILYYKRAP EAKNEFAFLE PFAEEVWYYL MLTQLIMTLL FVILARFSHH EWTKPNPADA DPDELENIWN NSNSFWLMIG SIMQQGCDIL PKGPPMRILS STWWFFTLMM VNAYVANLAA SLTNNMLPSE FDSLESLIEQ NKVKYGTLAG GSTSVFFSES NETEYKRAWN QMISFTPSAF TSSNKEGVDR VRKGNGSYAF LMETTSMSYN IERNCNLKQV GSQFSEKHYA LAVPLGAEYR SNLSVSLLQL SEKGELYNLK RRWWTPEEEI VCQIDVKADG DELSIIELSG VFVVLGAGVV VSFII

>BminIR17

NSYQEIFKMR FFLKRAFHIY FLMLVVIKAS SSLPPVIRVG AISSDMQDGP IDLAFKYAIH SINKDVSLLP ETKLTYDLQH VAANDSFHAY QKVCAQIESG IHAVFGPFNA ILGVHIHSIC DALDIPHMES RIHHNVANTE FSINLNPAKE YVNLAFEDII RYLNWTKTGI LYEKDFGVLQ LHKFGQMYDI ETYLRLVNPT TYVKILNELK DKEIHNLIID TNAANISILL KGILQLQMNE YKYHYLFTSF DIESFDLEDF KYNFVNITSF RLVDVGDVSV RNILKNMEAY NYDYNSKPTY YRKLRTIKTE SAVVYDSVHV FAIGLTSLEQ SLTLGVSNAS CASENPWDGG LSLINYINSV EWRGLTGPVQ FKEGQRVKFK LDLVKLREHS LVKVGEWTPQ TRLNITEPTL FFDGATMNVT LVVITILETP YVMMHYGKNY TGNERFFGFC VDILELIARH VGFDYILDLV PDRKYGAQDP FSGEWNGMVAQLMKYKADLA VGSMTITYAR ESVIDFTKPF MNLGISILFK VPSSPASRLF SFMNPLAYDV WLYALAAYFL VSFTIYIVAK LSPIEWRDKY PCDMKNPRIT NQFTLANSFW FTIGTLMQQG SDINPKSLST RIVSAIWWFF TLIIIASYTA NLAAFLTVER MITPIENAED LASQTEISYG TLESGSTMTF FRDSIIETYK KMWRNMENKK FIAFTSTYEE GIKRVNQGNY AFLMESTMLD YIVQRDCNLT QIGGLLDTKG YGIATPKGSP WRDKISLSIL ELQEKGNIQM LYDRWWKKAG HTCVRKSNKK QTKANALGLV NIGGVFVVLF VGIGVAASVA VFEFWYHYRT RNRESVNCEK QHKTVGSDMI EMALNAFDFA DKSDKENVEE IHTEHQHFSQ SHKQPWKHVT ACCCTKPAAQ RSLCGEMLDE FRYALRCMDS HRRPALKRRC PTCHLFKDIK EFIDVETRAS TNVRCSPLGS ASNDLKMHHT QGKSTTSSDY

>BminIR18

VL KTSLSEIING LKPRQLAILA APQFHFTKRH APLPASAEIP DDSQLEGMQM DIDDFIYQLH KLNFKSVIYD KADLFFKFVE DSLQGSIESV NLIFSAPYEL SARIQERRLS HRLSLFIFYW GAKHPPKANE VRFEEPMRAV VITRPRKKAF RIYYNQAVPD GVSNLRLVNW YDGDNLGLQK VPLLPNAATVYSNFNGRVFR VPVFHSPPWF WVSYENDSTN STMLDDYIDS TNDYNEFTEV NVTGGRDHCL LNLLAQHMNF QFVYIEAPGR TQGSLRNDDT GEENDTFTGG IGLLQNGLAD FLLGDVSLSW ERRKAVEFSF FTLADSGAFA THAPRRLNEA FAIIRPFKRD VWPYLILTVI FS

>BminIR19

EEPFQNVSCP LTTSMALVYD GVQLLAETFK HVMFRAVPLNCNDASSWDKG YTLVNYMKSL SLTGLTGEVK FDYEGLRTDF VLDVIELTMS GMQKIGEWKT EGGFFANRPP PKIVEVDQRS LVNKSFVVIT AISEPYGMLK ETPAKLEGND QFEGFGIELI EELGKKLGFT YTFRLQVDNK YGSFNPKTGK YDGMMLEIIE GRADMGITDL TMTSIREEGV DFTIPFMNLG IAILFRKPMK EPPKLFSFMS PFSGTVWMWL GIAYMSVSLT LFILGRISPT EWDNPYPCIE EPTELENQFS FPNCLWFSIG ALLQQGSELA PKAYSTRTVA SIWWFFTLIL VSSYTANLAA FLTIESLSSP IENAEDLANN KGGVKYGAKV GGSTFTFFQD AKYPTYQKMY EFMRDHPEYM TSTNAEGVDR VENENYAFLM ESTTIEYITE RRCSLTQVGS LLDEKGYGIAMRKNWPYRDMLSQAVLELQEQGVLTKMKTKWWKEKRGGGA CSDTSSEGGA VALELSNLGG VYLVLIVGSCFGVLVAFLEM VLGVKERSDE NKVSFKTELI EEFRFVMQCS GNTRPVKYPK NSSRSRSRSS RSRSHSRSSS KSSTLSVDSLPMDESKLHHI SEHTKHAK

>BminIR20

SR ENLQLENCCD MARPYRQSLS HFVATARCTR ITLIILVVLS FLQIADAQKT NVGLIYESNN PDMEKIFQIA IDKANEESGG TLELHGIAVA IESGNAFETS KKLCKMLRQN LVAVFGPTTD LAAKHAMSIC DAKELPFIDT RWDFGVQMPT VNLYPHASQL AVALKDLVVA LEWTDTFTII YETGEFLPTV NELLEMYATA GPTITVRRYE LDLNGDYRNV LRRIKNSGDY SFVVVGSMDT LPEFFKQAQQ VGLMTSDYRY IVGNLDLQTMDLEPFQHGDT NITGIRLVSP DAQGVQQLAK ALYETEEPFQ NVSCPLTTSM ALVYDGVQLL AETFKHVMFR AVPLNCNDAS SWDKGYTLVN YMKSLSLNGL TGEVKFDYEG LRTDFVLDVIELTMSGMQKV GEWTTENGFF ANRPPPKIVE QDQRSLVNKS FVVITAISEP YGMLKETSAK LEGNDQFEGF GIELIEELGK KLGFSYTFRL QVDNKYGSYN PKTGKYDGMM LEIIEGRADM GITDLTMTSI REEGVDFTIP FMNLGIAILF RKPMKEPPKL FSFMSPFSGT VWMWLGIGYL SVSLTLFILG RISPTEWDNP YPCIEEPTEL ENQFSFPNCL WFSTGALLQQ GSELAPKAYS TRTVASIWWF FTLILVSSYT ANLAAFLTIE SLSSPIESAE DLAVNKGGVK YGAKVGGSTF TFFQDAKYPT YQKMYEFMRD HPEYMTSTNA EGVDRVENDN YAFLMESTTI EYITERRCSL TQVGSLLDEKGYGIAMRKNWPYRDTLSQAVLELQEQGVLTKMKTKWWKEK RGGGACSDTS SEGGAVALEL SNLGGVYLVL IVGSCFGVLV ALLEMVLGVK ERSDENKVSFKTELIEEFRFVMQCSGNTRPVKYPKNSSRSRSRSSRSRSRSRSHSRSSSK SSTLSVDSLP MDETKLHHIS EHAK

>BminIR21

ESSEYLSILN GLMPFYGSDG PIINVLRYDL KLSGNFKAVL RRVRKSEEGH IVVVGSTPSV AELLKQAQQV GIMNDKYSYI IGNLDLQTFD LEEYKYSEAN ITGFRMFSPT QAIVQELISQ LEMDYNENNN NQIANGSCPI TLEMALTYDA VQVFAESTKN LVYRPQALNC SEQSNQVQAD GSTFKNYMRS INMQEKTITG PI

>BminIR22

DPLSEQPIRT LLHDLLYDAV HVFANALRNV SYSYQIRPPR VRCDFTEYEQ MQPWPMGRYI YRVMLATSGV NNTDYRTSEL

>BminIR23

NRMTVNVAPS VSMISKAYYS IINSNYEWDQ FTLIYETQTG LARLQDLMNI KPLDSEQIKI RYIEDYKSDL RVLWKEI

>BminIR24

KKTRLRPIDE IFQNQTLLPI LMYDAVVLFA NAARNIITKT RTYTKPQRRC EFDYPGRPWY IGRQIVNEMK SISEDDVEP

>BminIR25

DLEIFDLED FKYNGVNITA FRLVDVESQR YKEVIEQMQK LPHSGLDYIN EQPYIQAQSA LMFDSVYSVA AGLMELDRNH LLTWHNISCKNELAWKDGLS LYNYINSASM NGLTGRVHFS EGRRNIFQVD LLKLKREKIE KVGVWKPDVG VNITDSAAFY DTHTSNTTLI VMTRQEKPYV MVKRDISQTG NDRFEGFCID LLKAIATQVG FQYEIELVPD NMYGVFNPDT KVWNGIVREL MEKRADLAVA SMTINYVRES VIDFTKPFMN LGIGILFKVP TSQPTRLFSF MNPLAVEIWL YVLAAYVLVS LTLFVMARFS PYEWNNPHPC MKESDIVENQ FSVSNSFWFI TGTFLRQGSG LNPKAVSTRI VGGIWWFFTL IIISSYTANL AAFLTVERMI TPIEGASDLA EQSDISYGTL EGGSTMTFFR DSKIDTYQKM WQYMETRHSS VFVRTYEEGI KRVMEGNYAF LMESTMLDYA VQRDCNLTQIGGLLDSKGYG IATPKGSIWR DPMSLAILEL QEKGIIQILY DKWWKNTGDV CNRDEKSKES KANALGVENI GGVFVVLLCG LALAVVVAIL EFCWNSKQTL KLAETQTLCS EMTEELRYAT HCHESKQLQS LKRNSAKFPP DTTYVPADTR NGIPNSSGVH YNYFD

>BminIR26

DLEIFDLED FKYNGVNITA FRLVDVESQR YKEVIEQMQK LPHSGLDYIN EQPYIQAQSA LMFDSVYSVA AGLMELDRNH LLTWHNISCKNELAWKDGLS LYNYINSASM NGLTGRVHFS EGRRNIFQVD LLKLKREKIE KVGVWKPDVG VNITDSAAFY DTHTSNTTLI VMTRQEKPYV MVKRDISQTG NDRFEGFCID LLKAIATQVG FQYEIELVPD NMYGVFNPDT KVWNGIVREL MEKRADLAVA SMTINYVRES VIDFTKPFMN LGIGILFKVP TSQPTRLFSF MNPLAVEIWL YVLAAYVLVS LTLFVMARFS PYEWNNPHPC MKESDIVENQ FSVSNSFWFI TGTFLRQGSG LNPKDSKIDT YQKMWQYMET RHSSVFVRTY EEGIKRVMEG NYAFLMESTM LDYAVQRDCN LTQIGGLLDS KGYGIATPKG SIWRDPMSLA ILELQEKGII QILYDKWWKN TGDVCNRDEK SKESKANALG VENIGGVFVV LLCGLALAVV VAILEFCWNS KQTLKLAETQ TLCSEMTEEL RYATHCHESK QLQSLKRNSA KFPPDTTYVP ADTRNGIPNS SGVHYNYFD

>BminIR27

VDSPRGFTSR FMTNVWALFA VVFLAIYTAN LAAFMITREE FHEFSGLNDS RLVHPYSHKP SFKFGTIPYS HTDSTIHKYF KDMHHYMRQY NKTS

>BminIR28

ENMFWYVFGMFTNSLTFSGKYSWTSTQKSSTRLLIGSYWLFTIIITACYTGSIIAFVTLP AFPNTVDSVN DLLGLFFRVG

>BminIR29

EGFNLYPNPIDLARILYDIIHLFEWPRFIFLYESSDYLSILNGLMSFYVSDGPVIKVLRY DLKLNGNYKA VLRRVRKTED GHIVVVGSTS SVAELL

>BminSNMP1a

I TMKVQRHKLL IASVSAMLFG IIFGWVGFPK ILKTMIKKQV SLKPGTEIRD LWTQTPFPLH FYIYVFNITN PDEVMNGEKPNLQEIGPFVFDEWKDKYDLV DDPMEDSISF NMRNTFYFNE KDSKGLTGEE LITIPHPLIV PISVVVQRER AAMLDLVSKA INIVFAGQKA VITTKFMDVF FRGIYVDCSS PEFAAKALCT AFYTGEVKQA KQVNSTHFLF SFMENNNHTD GGRFTVCRGV KNVSKLGKVI RFGDEPTLDI WGGEECNEFI GTDSTIFAPF MTKEQGLWAF TPDLCRSFGA VFKRKSSYHG MPAMRYHMDL GDIKADPSLH CFCDDPENSE SCPPKGTMNL EPCVGAPIMA SMPHFYNADP SLLEEVNGLS PNEKDHAVFI DFELTSGTPF QAAKRLQFNL DMEPVEKIEP TKNLRKMIFP LFWVEEGVAL NKTFTNMLKY TLFLGLKFNS ALRWSLITMALVGLMSTCYL YYKKSDSIDI TVPPKAITEL TNKVEDVKPL PPGDKRPVPP IAEADLSNRR DTTNRF

>BminSNMP1b

KMFKKILIG SAIALVLGIF VGFVGFPKLL NKMIKGQLNL KPGSEPRKMW EKFPIAVNFS IYVFNLTNPD EVQNGGKPHV QEVGPFVFEE WKDKYDLEDF EDEDAVAYNM RNTFIFRPDL GLSGEDLIVM PHPLIQVMSI AVKRDKEALI HMIAEGIEAI FKPTTPFVRA PFMDIFFRGI DVDCSVDHFA VTAICLNFHT GAVKGAEKVN ATHFKFSLFG GANHTDAGRYKVARGVKVSHDIGRVLEFDD SDELSVWDGD ECNQFRGTDT TIFAPLLKPE EGLWSFAADL CRSLGAQFEK KTSYAGIPAY YYTIDLGDPK NDPDKHCFCR DYPDDCPPKG TMDLTLCNEA PLIVSLPHFF KADPKLVADV DGLNPQEEKH GVFIVFERIS GTPLSAAKRL QFSLSVMPVP EIEVMKNLRT LTMPLFWVEE AASLDKTWTD MLKKKVFLVI KINNIFKWMS TIFGALGLII SLYMLYGKNQ ITTTNVTPTT EINTIDKH

>BminSNMP1c

DVNKVLNVE EDVEIESQSV EVVHIHTRLY LRVKETLNAK IAKSLFPGSI PWYSTKMFHW SLFVSLLGLL IAALGAYCGW FLFPNMVDKK VEESVIIADG SEQYKRFVQL PQPLTFKVYI FNVTNAHKIQ QGAIPIVEEI GPYVYRQYRR KKVKHFSRDG SKISYVQDQH FEFDEEASAP YTQSDHIVVL NMHMNAFLQV FEREITDIFQ GFANRLNHRL NRTPGVRVLK RLMERIRGKR KSVLTIAEND PGLSLLLVHL NANLKAVFND PKSMFLDTTV REFLFDGVRF CINTNGIAKA ICNQIKEGGS KTIRELSDGS LAFSFFNHKN GTGNEVYEVH TGKGDAQRVL EIQKLDDSHN LQVWLNGSEG ETSMCNQING TDASSYPPFR KRGDSMYIFS ADICRSVQLF YQSDIQYQGI PGFRYSIGEN FINDIGPEHD NECFCVDKLA NVIKRKNGCL YAGALDLTTC LDAPVILTLP HMLGASNEYT KMIRGLRPDA KKHQTFVDVQ HLTGTPLQGG KRVQFNMFLK SINRISITEN LTTVLMPAIW VEEGIQLNSE MVAFFKKKLI NSLKTLNIIH WASICGGIGV AAICLIYYVI QRRKPEAEVA PLK

>BminSNMP2a

LKLKMFKKILIGSAIALVLGIFVGFIGFPKLLNKMIKGQLNLKPGSEPRQMWEKFPIALNFSIYVFNVTNPDEVQNGGKPRVQEVGPFVFEEWKDKYDLEDFEDEDAVAYNMRNTFIFRPDLGLSGEDLIVMPHPLVQIMAIAVKRDKEALINMISEGLQALFKPTTPFVSAPFMDIFFRGIDVDCSIDHFAAKAICLNFHTGAIKGAEKVNSTHFKFSLFGGANHTDAGRYKVARGVKVSRDIGRVLEFDDSDELSVWDGDECNQFRGTDTTIFAPLMKPEEGLWSFAADLCRSLGAEFEKKTTYAGIPAYYYTIDL GDPKNDPDKH CFCKDYPDDC PPKGTMDLTL CNEAPMIVSL PHFFKADPQL VADVDGLDPVEEKHGVFIVFERISGTPLSAAKRLQFSLSVMPVPEVEVMKNLRTLTMPLFWVEEAASLDKTWTDMLKKKVFLVIKINNVFKWMSTIFGALGLAISLYMLFGRNQITTTNVTPTTEAST
